# Seagrass Meadows: Prospective Candidates for Bioactive Molecules

**DOI:** 10.3390/molecules29194596

**Published:** 2024-09-27

**Authors:** Hazeena M. Ameen, Ayona Jayadev, Geena Prasad, Deepa Indira Nair

**Affiliations:** 1 Postgraduate Department of Environmental Sciences, All Saints’ College (Affiliated to the University of Kerala), Thiruvananthapuram 695007, India; hazeena248@gmail.com; 2 Department of Mechanical Engineering, Amrita Vishwa Vidyapeetham, Amritapuri 641112, India; 3 Department of Engineering Technologies, Swinburne University of Technology, Melbourne 3122, Australia; dnair@swin.edu.au

**Keywords:** seagrasses, biochemical composition, phytochemical analysis, antimicrobials, antioxidants, bioactive compounds

## Abstract

Seagrass meadows consist of angiosperms that thrive fully submerged in marine environments and form distinct ecosystems. They provide essential support for many organisms, acting as nursery grounds for species of economic importance. Beyond their ecological roles, seagrasses and their associated microbiomes are rich sources of bioactive compounds with the potential to address numerous human healthcare challenges. Seagrasses produce bioactive molecules responding to physical, chemical, and biological environmental changes. These activities can treat microbe-borne diseases, skin diseases, diabetes, muscle pain, helminthic diseases, and wounds. Seagrasses also offer potential secondary metabolites that can be used for societal benefits. Despite numerous results on their presence and bioactive derivatives, only a few studies have explored the functional and therapeutic properties of secondary metabolites from seagrass. With the increasing spread of epidemics and pandemics worldwide, the demand for alternative drug sources and drug discovery has become an indispensable area of research. Seagrasses present a reliable natural source, making this an opportune moment for further exploration of their pharmacological activities with minimal side effects. This review provides a comprehensive overview of the biochemical, phytochemical, and biomedical applications of seagrasses globally over the last two decades, highlighting the prospective areas of future research for identifying biomedical applications.

## 1. Introduction

Marine ecosystems are the largest aquatic ecosystems with a rich biodiversity. It consists of a wide variety of organisms that exhibit a unique gene pool and genetic characteristics due to the ecosystem’s high physical, chemical, and biological diversity. Marine organisms take part in various critical processes that directly or indirectly affect the health of marine ecosystems [1]. A substantial proportion of the human population benefits from the marine environment for their livelihood and survival. Along with the marine fauna, the marine plants also provide many services, and marine angiosperm is a prominent group.

**Figure 1:**
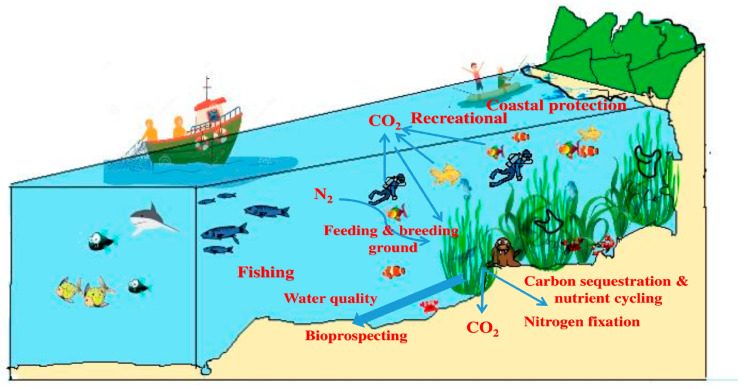
Societal benefits of seagrasses—ecosystem services, including the bioactive potential of seagrasses. The figure shows the various ecosystem services rendered by seagrasses, listed as follows: carbon sequestration, nutrient cycling, the maintenance of water quality, the protection of coasts, recreational values, the provision of feeding and breeding grounds, fishing, and recreational values.

Seagrasses are the only blooming plants in the marine realm, adapted to complete their lifecycle as wholly or partially submerged flora. Coastal ecosystems are often overseen by coral reefs and mangrove ecosystems. It is believed that seagrasses were once terrestrial vegetation and later inhabited the shallow waters of the sea [2]. There are only 72 odd seagrass species, which comprise less than 0.02% of angiosperms. Seagrasses have morphological, physiological, ecological, and genetic adaptations for submerged growth, such as the marine dispersal of seeds, the internal movement of gasses, aquatic pollination, and epidermal chloroplasts. Generally, they inhabit intertidal and mid-tidal shallow zones with more light exposure. They are monocotyledonous plants that come under four families: Posidoniaceae, Zosteraceae, Hydrocharitaceae, and Cymodoceaceae [3]. Seagrass beds provide valuable services as nourishing and breeding places for various marine lifeforms, including sea turtles, dugongs, and fishes [4]. They are also considered the “lungs of the sea” due to their rich oxygen generation through photosynthesis. The released oxygen purifies the ocean environment and contributes to the dissolved oxygen that other marine organisms can use. These outstanding floral groups influence their environment’s prevailing physicochemical and biological conditions [5] and are crucial in combating climate change [6]. Seagrass beds help stabilize loose sediments and filtrate solid pollutants, improving water quality [7]. As per the Wealth of India Division (2013) [2], one hectare of seagrass meadow absorbs nutrients equivalent to treated effluent from 200 people. It sequesters approximately 33 grams of carbon per square meter per year. This quantity of carbon corresponds to automobile emissions when traveling 2500 km. One hectare of seagrass meadow is also responsible for 12% of locked organic carbon, which reduces greenhouse gasses. Almost every nation with seagrass resources, including India, uses seagrasses for their nutritional and medicinal properties (Figure 1).

Seagrasses have surface microbial communities inhabiting their roots, leaves, rhizomes, and endophytes, and many species act as the source for potential novel bioactive compounds [8,9,10,11] in addition to the bioactive molecules produced by the seagrasses themselves. A recent review article [12] highlights the importance of seagrasses by detailing the pharmacological potential of *Thalassia hemprichii* and *Thalassia testudinum*, their fractionation processes, phytochemical constituents, bioactive potential, pharmacological capabilities, and their action mechanisms. Marine lifeforms will adapt to immoderate environmental conditions, including higher pressure and salt concentration, minimal nutrient concentration, low-level but consistent temperature, limited sunlight, and low oxygen. Thus, marine organisms require and possess distinct characteristics, distinguishing them from terrestrial organisms [13].

The emergence of novel diseases, changes in pathogenicity, and antibiotic and drug resistance necessitate an alternative source or novel bioactive compounds [14]. This scenario leads to the necessity of bioprospecting. Even though numerous investigations have focused on the bioprospecting of many marine species [15,16], information regarding the bioactivity of marine angiosperms is still scarce. The coastal population uses seagrasses for healing skin diseases, fever, muscle pains, stomach aches, and wounds, and is also used in children as tranquilizers for ray stings [17]. In folk medicines, seagrasses are used widely for various ailments. However, the scientific approach to studying bioactive molecules from seagrasses is still in its infancy. This review aims to familiarize the biochemical composition of seagrasses and the current state of seagrass bioprospecting, and to make an inventory of bioactive compounds isolated from seagrass in the last two decades. The analysis of the available literature finds some gaps in the research on seagrasses. While extensive study exists on the phytochemical and biochemical characteristics of seagrasses, inquiry on the functional heterogeneity and roles of seagrass-related microbiomes remains sparse. The interactions between seagrasses and their microbiomes in the biosynthesis of bioactive compounds require investigation. There is an apparent shortage of research linking bioactive compounds to their specific ecological functions. Additionally, understanding how environmental stressors such as climate change and pollution affect seagrass bioactivity is important. Moreover, there is a lack of detailed research that combines genetic, metabolomic, and ecological data to emphasize the ecological effects of bioactive compounds and their biosynthetic pathways.

## 2. Methodology of Literature Retrieval

**Figure 2:**
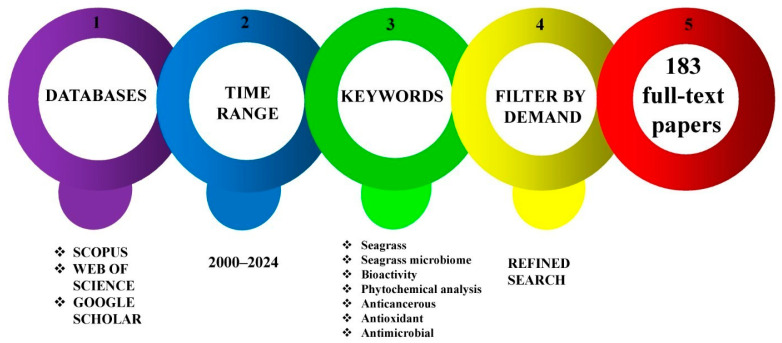
Bibliographic search pathway. The figure shows the details of the databases used for the literature study using the set of keywords from 2000 to 2024. After applying filters, 183 full-text manuscripts were used to write this manuscript.

**Figure 3:**
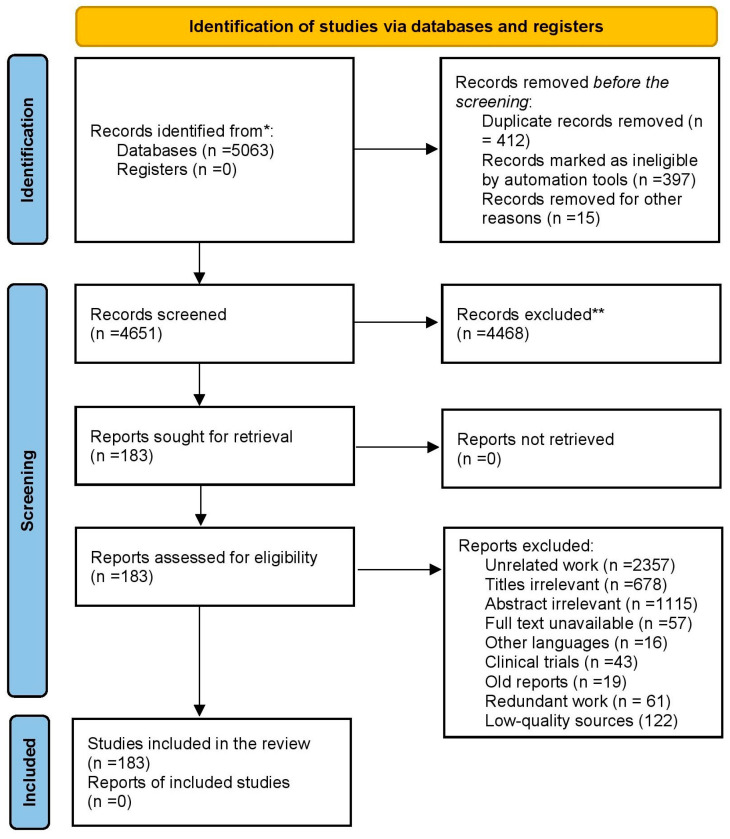
PRISMA flow diagram (Haddaway et al. [18]). The figure shows the methodology adopted for selecting manuscripts to prepare this review through identification, screening and inclusion. * Scopus, ISI Web of Science, and Google Scholar. ** 4468 records were excluded due to various reasons unrelated work, irrelevant titles, irrelevant abstract, unavailability of full texts, reports in other languages, human trials, old reports, redundant work and low-quality sources.

A thorough and deliberate evaluation of the literature has been carried out by exploiting the reputable scientific data repositories “Scopus” (https://www.scopus.com, accessed on 30 April 2024), “ISI Web of Science” (http://apps.webofknowledge.com, accessed on 15 August 2024), and Google Scholar (https://scholar.google.com, accessed on 2 August 2024). All significant and important published research on the subject has been examined and sorted based on demand (Figure 2). Keywords have been applied to the following Boolean logic: [(Bioactivity*seagrass) * OR seagrass distribution* OR nutritional analysis of seagrass* phytochemical analysis of seagrass* OR antimicrobial activity * OR anticancerous activity * OR antidiabetic activity * OR anti-inflammatory activity * OR seagrass ecosystems*] AND [(Potential activity* seagrass microbiome) OR larvicidal activity* OR antibacterial* OR antiviral* OR antifungal* OR seagrass ecosystem*, OR seagrass endophytes*, OR bioprospecting*]. From the data repositories referenced, a total of 5063 records were obtained. Using the Zotero reference manager, 397 duplicate records were identified and removed. Additionally, fifteen records were manually excluded due to lack of clarity. Of the remaining 4651 records, 4468 were removed for various reasons, 2357 were unrelated works (though the search using the keywords showed the manuscripts, they were not in the bioprospecting genre), 678 had irrelevant titles (the titles could not be related to the manuscripts under consideration), 1115 had irrelevant abstracts (the abstracts were not of the study area under consideration), and 318 had either no full text available or were in other languages. The excluded 318 articles also reported human trials, old reports, redundant works, and low-quality sources. A total of 183 whole-text publications were reviewed; these serve as the investigation’s output (Figure 3). This manuscript complies with the PRISMA guidelines [18]. After each study was evaluated, a final screening was conducted to ensure that all articles considered directly influenced seagrass bioprospecting.

## 3. Trends in Seagrass Research

**Figure 4:**
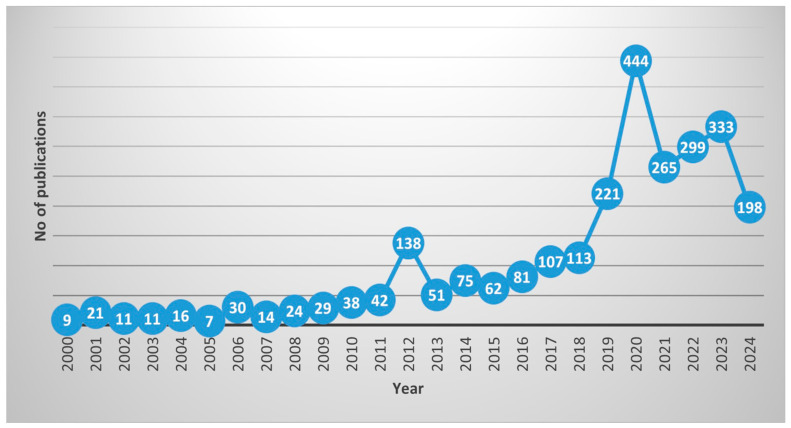
Line graph showing the number of publications through the years 2000–2024. The figure shows a surge in publications related to seagrasses during the years 2012 and 2020.

The literature was examined methodologically by compiling the available literature on seagrasses, bioprospecting, and bioactivity studies, as explained in the previous section [18]. The distribution of publications through the years 2000 to 2024 is shown in the line chart (Figure 4). The vertical axis displays the total number of publications, and the horizontal axis represents the years. The line graph shows an upward trend between 2000 and 2012. With a few notable exceptions, the number of publications steadily increased from 9 in 2000 to a maximum of 2020 in 2020, with 444 publications in that year. This upward trajectory denotes a significant period of increased research output. After 2020, though, the trend drastically changed, with an abrupt decline to 265 publications in 2021. After this decline, there is a modest comeback in 2022 and 2023, with 333 publications on average. Based on the data, there are significant dynamics in seagrass research, and after 2020, there seems to be a shift in the priority areas of research. 

In the current scenario of emerging pandemics, the relevance of bioprospecting natural resources has become increasingly evident. The study on the area again found a hike, with 333 articles published during 2023 and 198 articles published in 2024 within eight months. As novel pathogens and resistant strains challenge existing medical treatments, there is a demand to explore and tap the therapeutic potential of natural compounds [14]. Bioprospecting, particularly in underutilized resources like seagrasses, offers a promising avenue for discovering new bioactive substances with antimicrobial, antiviral, and anti-inflammatory properties. By looking into the rich chemical diversity of natural marine resources, researchers can identify novel agents that could create effective aid and preventive measures against emerging infectious diseases.

**Figure 5:**
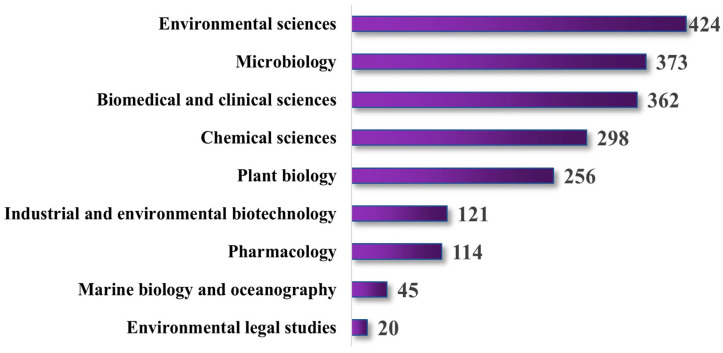
Bar graph showing seagrass bioprospecting publications in different subject domains. The bar graph shows that the publication on seagrasses is highest in the environmental sciences field and minimum in environmental legal studies.

The distribution of publications on seagrass bioprospecting from various subject domains [15,16] during the years 2000–2024 is shown in Figure 5. The vertical axis indicates the number of publications. In contrast, the horizontal axis represents studies from different subject domains, such as environmental sciences, microbiology, biomedical and clinical sciences, chemical sciences, plant biology, industrial and environmental biotechnology, pharmacology, marine biology and oceanography, and environmental legal studies. The maximum number of publications came from the domain of environmental Sciences, with a total of 424 articles, making it the most researched area. Subsequently, the ‘microbiology’ domain shows a noteworthy number of publications of 373. The other fields with the highest representation were biomedical and clinical sciences, chemical sciences, plant biology, industrial and environmental biotechnology, and pharmacology, with 362, 298, 256, 121, and 114 publications, respectively. The fields with the lowest number of publications were “marine biology and oceanography” and “environmental legal studies,” with 45 and 20 publications, respectively.

### 3.1. Seagrasses: The Marine Angiosperms

As the only submersed marine angiosperms with a buried root, the rhizome system of seagrasses is specifically adapted to thrive in marine habitats. Even though they emerged early in the angiosperm evolutionary timeline, Burt (2001) [19] observes that their evolutionary path differs from their terrestrial counterparts, showing a relative stasis. Seagrass beds are vital to maritime countries worldwide because of their products and services [20,21,22]. This comprises significant contributions like the filtration and cycling of nutrients [23], the retention of sedimentary carbon [24], and the significant support for worldwide fishing industries [25,26]. Even though seagrasses only constitute a small part of the marine realm, they render a wide array of ecosystem services. Seagrass serves as a protective home for a diverse number of marine lifeforms, a food source and habitat for several fish species, including *Egretta garzetta*, a seasonal migratory bird, as well as for threatened species like endangered green turtles (*Chelonia mydas*), seahorses, and dugongs (*Dugong dugon*) [27].

Several studies show the critical ecological roles of seagrasses in coastal ecosystems, including providing habitat for marine life, stabilizing sediments, and mitigating coastal erosion. However, only a few studies are reported detailing the bioactive potential of seagrasses [28,29]. Seagrasses are abundant reserves of bioactive products with potent pharmaceutical and nutraceutical applications. Bioprospecting involves systematically screening seagrass extracts to identify bioactive compounds with biomedical potential. Such compounds may possess anti-inflammatory, antimicrobial, anticancerous, or antioxidant activities, offering potential benefits for human health [30,31,32,33,34]. A nutritional analysis is essential for identifying and quantifying these bioactive compounds. The bioprospecting of seagrasses can promote their conservation and sustainable use by demonstrating their economic value beyond ecological values and traditional uses. Identifying novel applications for seagrasses can incentivize their protection and restoration, enhancing the overall wellness and durability of coastal ecosystems.

### 3.2. Nutritional Analysis of Seagrass

Seagrasses have been traditionally consumed by coastal communities in various parts of the world. Understanding their nutritional composition through analysis is crucial for assessing their potential as a food source and determining their contribution to human nutrition and health. Nutritional analysis can identify essential nutrients, such as proteins, lipids, vitamins, and minerals in plants, essential for overall well-being [35]. Compared to other marine organisms like seaweeds and seagrasses, vascular plants usually contain higher concentrations of complex carbohydrates, proteins, and lipids [36]. They also include vitamins, zinc, and iron, among other vital micronutrients. Many compounds found in seagrass have beneficial nutritional and calorific value. Even though they are high in nutrients, limiting factors like tannin and phenol preclude them from being used in human diets. In the recent two decades, there has been a surge in research into their biochemical composition and potential for use as a food source [37,38]. Several macrophyte species like *Halymenia dilatata*, *H. maculata*, *Halophila ovalis*, and *Enhalus acoroides* were loaded with at least one mineral (e.g., P, Si, Zn, K, S, or V). Compared to some common edible seaweeds and terrestrial plant sources, the mineral concentrations were significantly greater in many tested species [39].

**Figure 6:**
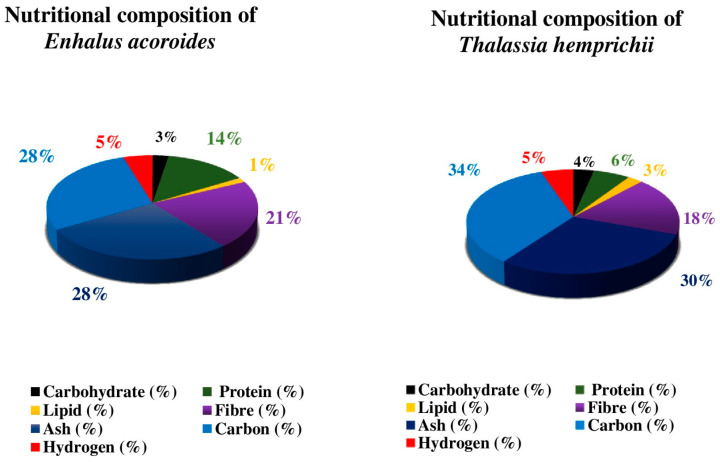
Nutritional composition of *Enhalus acoroides* and *Thalassia hemprichii* [35,36,40,42]. The most significant component of *E. acoroides* and *T. hemprichii* is ash and the least is lipid.

**Figure 7:**
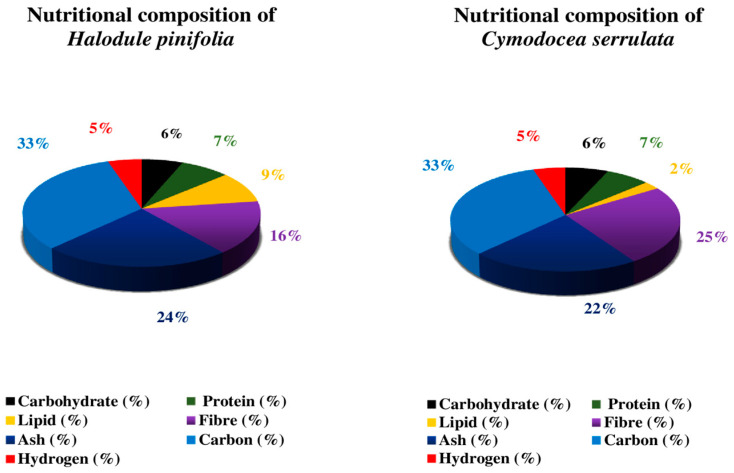
Nutritional composition of *Halodule pinifolia* and *Cymodocea serrulata* [35,36,40,42]. The most significant component of *H. pinifolia* and *C. serrulata* is ash and the least is carbohydrate in *H. pinifolia* and lipid in *C. serrulata*.

**Figure 8:**
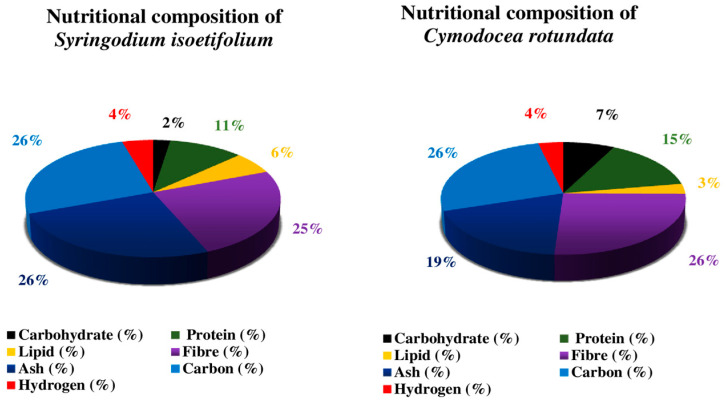
Nutritional composition of *Syringodium isoetifolium* and *Cymodocea rotundata* [35,36,40,42]. The most significant component of *S*. *isoetifolium* is carbon. In *C. rotundata*, carbon, and fiber constitute the major bulk of the plant, and the most negligible content in *S. isoetifolium* is protein, and in *C. rotundata* is lipid.

The pigment, biochemical composition, and secondary metabolites of eight species (*Enhalus acoroides*, *Halophila ovalis*, *H. beccarii*, *Cymodocea rotundata*, *C. serrulata*, *Halodule pinifolia*, *H. uninervis*, and *Syringodium isoetifolium*) of seagrasses were studied by the author. The results show a distinct seasonal fluctuation in chlorophyll concentration, with the lowest measure in the monsoon season and high carbohydrate and protein content in the summer [40]. Because seagrass has a large amount of carbs, using a carbohydrate-rich plant source to compensate for the protein-sparing effect in the diet could be a viable option. Micronutrients (Zn, Fe, Ni, Cd, Cu, and Cr) and macronutrients (P, K, Ca, Mg, and Na) are found in leaves of seagrass like *H. uninervis*, *C. serrulata* and *S. isoetifolium*. Carbohydrate, protein, and lipid composition were high in *Cymodocea serrulata* and *H. uninervis* but low in *S. isoetifolium* [41]. Figure 6, Figure 7 and Figure 8 show the results of another investigation that determined the biochemical composition (carbohydrate, protein, ash, phenol, flavonoids, tannin, and lipids) of six distinct seagrasses, *Thalassia hemprichii*, *Enhalus acoroides*, *Halodule pinifolia*, *Cymodocea serrulata*, *Syringodium isoetifoilum*, and *Cymodocea rotundata* [42]. In another study on the biochemical composition of seagrass *S. isoetifolium*, the carbohydrate (37.77 ± 0.27%) and ash (29.005 ± 0.455%) were found to be more significant when compared to the protein (15.92 ± 0.81%), lipid (5.1 ± 0.6%) and moisture (1.75 ± 0.15%). Thus, it indicates that *S. isoetifolium* is a good source of carbohydrate and protein [43].

### 3.3. Phytochemical Analysis

Phytochemical screening not only assist in identifying the more dominant components of plant extracts, but it also helps search for bioactive compounds that can be utilized to produce valuable drugs. Numerous studies illustrate the phytochemical components found in various marine life forms, but there is very little data on similar aspects connected to seagrasses, mainly from India [44]. These phytochemical compounds can be extracted with various solvents such as ethanol, acetone, hexane, and methanol. Phytochemicals like terpenoids, alkaloids, phenols, quinone, steroids, flavonoids, and glycosides were found in *Cymodocea serrulata* extracts, according to preliminary phytochemical analysis. The viability and existence of *C. serrulata* in marine waters could be due to these phytocompounds, which are an essential part of the defense system [45]. A phytochemical characterization of the methanolic fraction of *Enhalus acaroides* through liquid chromatography–mass spectrometry (LC-MS) identified the three kaempferol derivatives, like flavones, apigenin, luteolin, and azelaic acid [46]. According to another study, seagrass leaves have a higher phenolic content than roots and rhizomes, and there were differences in tannin concentrations among different species [47]. A pair of interesting prenylated flavon-di-O-glycosides from *C. nodosa*, labeled as cymodioside A and B, were discovered by phytochemical screening [48]. Coumarins, phenols, flavonoids, quinones, saponins, proteins, sterols, sugars, free amino acids, and terpenoids were discovered in aqueous methanolic extracts of six seagrasses (*Halodule pinifolia*, *Enhalus acoroides*, *Syringodium isoetifolium*, *Cymodocea serrulata*, *C. rotundata*, and *Thalassia hemprichii*). All six seagrasses lacked alkaloids and glycosides, while *E. acoroides* and *S. isoetifolium* lacked quinone [49]. According to Setyoningrum et al. [50], the phytochemical analysis revealed that *E. acoroides* extract included various bioactive components, including flavonoids, alkaloids, tannins, saponins, and steroids, and have some biological activity. When considering other fractions, the methanol fraction of *S. isoetifolium* displayed the most phytochemical components. Flavonoids, steroids, proteins, glycosides, alkaloids, and phenolic substances were found in methanol and acetone extracts of *S. isoetifolium*. Tannin and terpenoids were not present [43]. Phenols, tannins, and flavonoids were some of the phytocompounds in the chloroform–ethanolic (9:1) leaf fraction of *E. acoroides*. The most common functional groups discovered were phenols, benzenoid compounds, secondary amines, fatty acids, alkanes, and lipids [51]. Flavonoid, triterpenoid, terpenoid, steroid, tannin, and saponin were revealed to be active compounds in a recent study, with results varying from 3.21 to 8.63% for ethanol extracts and 0.21–0.76% for n-hexane extracts of *E. acoroides* [52]. In a recent study using Diaion^®^ HP-20 column chromatography, medium-pressure liquid chromatography (MPLC), and ODS column chromatography, a polyphenol substance MP-1 was refined from a 70 percent aqueous methanol fraction of *Phyllospadix japonica* and determined as rosmarinic acid (molecular weight-360 and molecular formula-C_18_H_16_O_8_) using electrospray ionization (ESI)-mass and nuclear magnetic resonance spectroscopic analysis [53]. In a recent study, the phytochemical components of *C. serrulata* were assessed. The existence of glycosides, flavonoids, steroids, phenolic compounds, tannins, carbohydrates, and ashes was identified. Using GC-MS analysis, metabolic profiling was carried out, and it uncovered the existence of 104 bioactive compounds in *C. serrulata*, including phytol, butanoic acid, heptadecane, diphenylamine, benzophenone, 2-hydroxyl-ethyl ether, benzene, octadecanoic acid, dotriaconate, vanillin and decane, all of which had significant roles in bioactive potential [54].

Essential metabolites like carbohydrates and proteins were discovered during the phytochemical analysis of *Enhalus acoroides*, as well as secondary metabolites like alkaloids, saponins, phenolics, monoterpenes, tannins, flavonoids, and sesquiterpenes [55]. Flavones from *Thallasia hemprichii* were found to be methoxy flavones or sulfated flavone glycosides [56]. Seven flavone glucosides were identified by Bitam et al. [57], as well as two malonylated flavone glucosides. Phenols and saponins were detected in significant concentrations in the phytochemical investigation of *Halodule ovalis* [58]. Phytochemical and proximate examinations were conducted on the seagrass *H. pinifolia*, and the outcome showed that the methanolic fraction of the seagrass *H. pinifolia* contained a variety of active secondary metabolites with remarkable bioactive potential [59].

### 3.4. Bioactivity of Seagrasses

**Figure 9:**
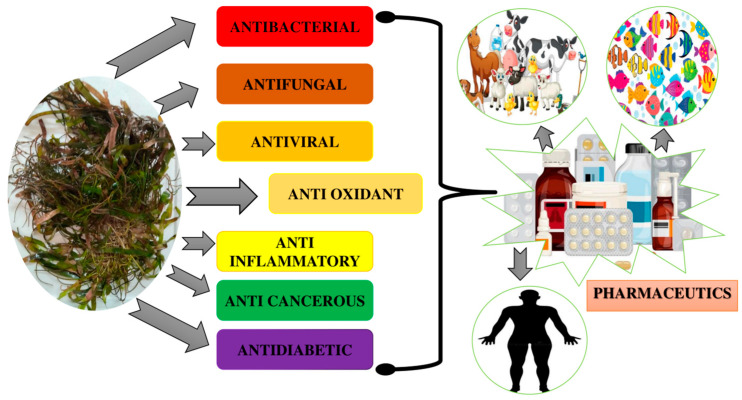
The bioactive potential of seagrasses. The figure shows the various bioactive potentials of seagrasses, which can be applied in various fields.

Studying the marine environment to discover various complex and novel chemical entities is a new trend in drug development from natural sources. It provides new lead compounds for treating several diseases, including diabetes, Acquired Immuno Deficiency Syndrome (AIDS), inflammatory conditions, cancer, arthritis, malaria, and a wide range of microbial infections [60]. In indigenous medicines, seagrasses have been used for many therapeutic purposes, including managing fevers, muscle aches, wounds, stomach issues, and skin conditions. They are also utilized as tranquilizers for infants and as a cure for various types of ray stings [61]. According to the existing literature and folk medicinal knowledge, seagrass possesses potent bioactivities (Figure 9).

**Figure 10:**
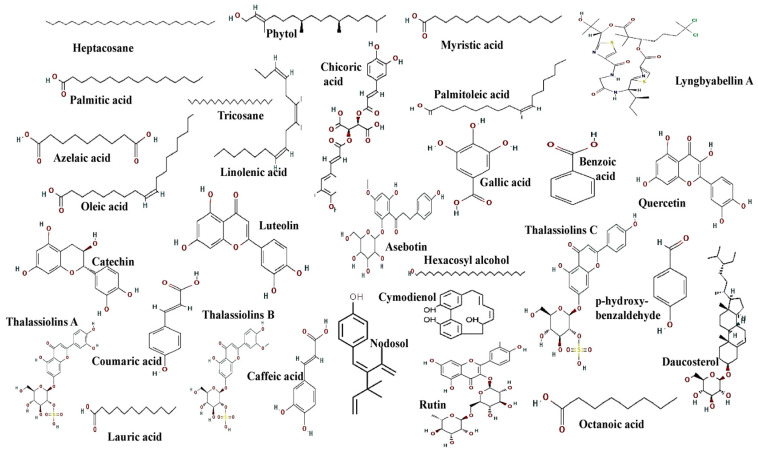
Structures of bioactive compounds isolated from seagrasses. This figure shows the chemical structure of bioactive compounds isolated from seagrasses by various researchers.

For the past fifty years, natural products from marine ecosystems have caught the interest of scientists worldwide. Compared to terrestrial products, marine lifeforms are a remarkable source of new bioactive compounds with a broader scope of structural and chemical characteristics [62]. Therefore, they are an excellent resource for determining and synthesizing bioactive compounds in marine organisms, and seagrass is an exceptional candidate. It is well known that seagrasses generate an ample range of metabolites, which serve as shields when they are under stress. Several varieties of seagrass provide biologically active metabolites, which include polyphenols, terpenoids, and halogenated compounds. These molecules have been shown to exhibit anticancer, antimicrobial, anti-inflammation, antidiabetic, antioxidant, cytotoxic, and anti-aging abilities. Numerous secondary metabolites produced by seagrasses improve the pool of novel and efficient medications for treating various illnesses. The bioactive characteristics of seagrasses, such as their anti-tumor, antimicrobial, and antioxidant properties, are primarily present due to these secondary metabolites [63]. Table 1 summarizes the bioactive compounds separated from various seagrasses, and Figure 10 depicts their structures. 

Table 2 summarizes the bioactivity of seagrasses. The following section discusses the antibacterial, anti-inflammatory, anticancer, antiviral, antioxidant, and tranquilizer properties of seagrass and their potential prospects.

Seagrasses are known for their diverse range of bioactive compounds, which are essential to their ecological roles and have great potential for various uses. Quercetin and kaempferol are seagrasses’ most common flavonoid compounds [70,73]. Kaempferol shows antioxidant and anti-inflammatory effects, along with potential cardiovascular benefits. Quercetin is well-known for its antioxidant, anti-inflammatory, and antiviral qualities. Additionally, phytol and other phenolic acids, such as coumaric and caffeic acids, are commonly found in seagrasses [64,65,68,81]. Seagrasses are consistently rich in essential vitamins (A, C, E, and K) and essential minerals (Ca, Mg, K, and Fe), all of which add to their nutritional and therapeutic value [40,41]. The literature suggests that the best extraction conditions for seagrass are usually achieved by using solvents such as methanol, ethanol, or chloroform, keeping the temperature between room temperature and 60 °C, and using techniques like maceration or Soxhlet extraction [85,86,87]. Depending on the need for extraction, the solvent-to-sample ratio ranges from 1:10 to 1:20 (*w*/*v*), and the extraction time can be about 3 to 5 days [72,74]. Particle size and pH changes may also improve the effectiveness of extraction. Extraction conditions must be modified to the particular objectives of the research or application, as each study may differ depending on the targeted compounds and seagrass species. Table 2 summarizes the bioactivity of seagrasses. The following sections discuss the antibacterial, anti-inflammatory, anticancer, antiviral, antioxidant, and tranquilizer properties of seagrass and their potential prospects.

### 3.5. Anti-Infectious and Antimicrobial Potential (Antibacterial, Antifungal and Antiviral)

Antimicrobial agents are substances that destroy pathogenic organisms while also limiting their development. The majority of microorganisms are highly immune to current antimicrobial medicines. As a result, many microbial infections necessitate using a novel agent. Seagrass-derived marine fungi have various biochemical potential and anti-infective and anti-tumor effects [10]. Of the three seagrasses (*Halodule pinifolia*, *Halophila stipulacea*, and *Cymodocea serrulata*) screened for antibacterial activity, *H. pinifolia* and *H. stipulacea*, outperformed *C. serrulata* in controlling the growth of human pathogens and also in controlling their spread. Methanol and chloroform fractions suppressed the development of all infectious agents among the solvents tested, with methanol fraction being the most involved [68]. Analyzing the EtOAc extract of the Egyptian seagrass *Thalassodendron ciliatum* (Forsk.) den Hartog, a novel dihydrochalcone diglycoside was isolated. It was recognized as 6′-O-rhamnosyl-(1‴ → 6″)-glucopyranosyl asebogenin, for which a name, Thalassodendrone, was proposed. The newly isolated compound’s anti-influenza A virus activity was assessed, and the results showed that its cytotoxic concentration and inhibition dose concentration were CC_50_—3.14 μg/mL and IC_50_—1.96 μg/mL, respectively [125].

The antibacterial potential of the three different seagrasses (*Cymodocea serrulata*, *Halophila ovalis*, and *Zostera capensis*) was examined using six different solvents against some human pathogens. The result shows that ethyl acetate extract of all three seagrass extracts was active against all Gram-positive and most Gram-negative pathogenic organisms studied, including *Bacillus cereus, Micrococcus luteus, Bacillus subtilis, Staphylococcus aureus,* and *Salmonella typhimurium*, with methanolic extracts active against *Staphylococcus aureus*, *S. typhimurium*, *M. luteus* and *Salmonella paratyphi*. Compared to other solvents, ethyl acetate and methanol extracts displayed the most activity against most pathogens [126]. Human bacterial pathogens such as *S. aureus*, *Shigella dysenteriae*, *S. boydii*, *Vibrio cholerae*, and *S. paratyphi* were found to have the most robust growth inhibitory activity in *H. pinifolia* and *C. rotundata* [49]. According to earlier research, seagrasses generate secondary metabolites that act as a suppressor against marine pathogens. With MIC values varying from 5 to 8 μg/mL, isoscutellarein and its glycosylated derivatives that were extracted from *Thalassia hemprichii* demonstrated antifungal activity against *Aspergillus niger* and *Candida albicans* [127]. The cyclohexane extract of *Posidonia oceanica* showed excellent antibacterial activity. *Pseudomonas aeruginosa*, *S. aureus*, *Klebsiella pneumoniae*, *S. typhi*, and *Escherichia coli* were all inhibited by the extract [128]. Compared to infectious microbial (fungi and bacteria) species, six solvent crude fractions of *Thalassia hemprichii* were tested for antimicrobial properties. The acetone crude fraction of *T. hemprichii* possesses high antibacterial activity, and pathogenic fungal species are inhibited by ethanol crude extracts [129]. *Syringodium isoetifolium* methanol and ethanol extracts have the highest antimicrobial activity against *E. coli*. Also, the extracts of *S. isoetifolium* suppressed the growth of bacteria such as *Psuedomonas aerunginosa*, *Bacillus cereus*, *Salmonella enteritidis*, *Staphylococcus aureus* [130] and saphrophytic fungus in silkworms [131]. *Thalassia testudinum* possesses an excellent series of HIV integrase inhibitors, which were detected and isolated [69]. The antiviral properties of polyphenol complex derived from the Zosteraceae family involve the direct deactivation of the tick-borne encephalitis (TBE) virus by the inhibition of its multiplication at an early stage, which consequently indicates a decline in the virus concentration [132].

According to a study by Setyoningrum et al. [50], *Enhalus acoroides* ethanol extract had antibacterial activity against *Staphylococcus aureus*. Methanol and the n-hexane fraction of *E. acoroides* has antibacterial action against *Staphylococcus aureus* with a bland zone diameter of 5.9 mm and 5.6 mm. Alkaloids, flavonoids, saponins, and steroids are found in methanol extract, while steroids and flavonoids are found in the n-hexane extract of *E. acoroides* [32]. The antimicrobial activity of the *E. acoroides* extract was assessed by using two different methods, the diffusion method and the minimum microdilution concentration method, against *Staphylococcus aureus* ATCC 6538, *Candida albicans* ATCC 10231 and *Escherichia coli* ATCC 8739. At an MIC of 500 ppm, the ethyl acetate fraction of *E. acoroides* inhibited the growth of *Escherichia coli* ATCC 8739, *Candida albicans* ATCC 10231, and *S. aureus* ATCC 6538 [31]. A recent study evaluated the antimicrobial activity of chloroform extract of seagrass *Cymodocea serrulata* against the following five pathogenic bacteria: *Vibrio parahaemolyticus*, *Vibrio harveyi*, *Vibrio alginolyticus* and *Klebsiella pneumoniae.* The antimicrobial properties were antagonistically effective against all infectious strains, where the highest inhibitory effect was observed against *V. parahaemolyticus* with a zone formation of 26 ± 0.08 mm. In contrast, the lowest inhibitory activity was seen against *V. alginolyticus* with a zone formation of 20 ± 0.04 [54].

### 3.6. Antioxidant and Anticancerous Potential

Antioxidants can help to protect the biological system from oxidative harm. Synthetic antioxidants have more side effects, whereas natural antioxidants are more effective at scavenging free radicals and have few side effects. The antioxidant property of phenolic compounds in seagrasses (*Cymodocea rotundata*, *Syringodium isoetifolium*, *Thalassia hemprichii*, *Enhalus acoroides*) was discovered. The ability to scavenge free radicals shows that it has antioxidant potential [133]. *C. rotundata* leaf and rhizome extracts serve as a prospective point of antioxidant compounds, with caffeic acid and coumaric acid being the most abundant compounds. This paves the way for their use as a multipotent antioxidant in the nutritional and biomedical industries [134]. The antioxidant potential of *E. acoroides* is primarily because of phenolic material, which was the main reason for the seagrasses’ antioxidant capability [68]. Methanol extracts of *Halodule pinifolia* seagrass have more potent antioxidant activity than ethyl acetate and n-hexane extracts [135]. The antioxidant and enzyme-inhibitory properties of *S. isoetifolium* were evaluated, and the existence of phenolic compounds may justify the observed biological activities [42]. The antioxidant potential of the methanolic extracts was in the order of *H. pinifolia* > *H. ovalis* > *S. isoetifolium*. Seagrass extracts’ antioxidant functions can be due to their ability to scavenge free radicals. Moreover, phenolic compounds are accountable for seagrass extracts’ antioxidant function [136]. The ethanol extract of *C. serrulata* has the highest free radical scavenging activity and contains many total phenols and flavonoids. The ethanolic fraction of *C. serrulata*’s efficient free radical scavenging and reducing properties demonstrate its fantastic antioxidant potential. Thus, it can cure different free radical-mediated diseases [45]. *C. serrulata* methanolic extract has antioxidant and cytotoxic properties. Since the sum of the compounds was proportional to the 2,2-diphenyl-1-picrylhydrazyl scavenging activity, the results showed that phenols and flavones may have played a significant role in the DPPH assay. As a result, the methanolic extract of *C. serrulata* can help prevent or treat such diseases, reducing the severity of oxidative stress-related diseases [137]. Recently, fibroblast cell line HS-68 was used to assess ethanolic extracts from *Posidonia oceanica* leaves in vitro while exposed to UV-induced oxidative stress. This study shows that antioxidant compounds in the ethanolic extract of *P. oceanica* are beneficial because pre-treating cells with Gd-E4 extracts significantly shield against oxidative damage and UV-induced mortality [138].

The bioactivity of Red Sea seagrass, *Enhalus acoroides* (L.f.) Royle, as an antioxidant and anticancer agent, was compared in a recent study. When tested on the normal cell line HSF, the leaves, roots, and rhizomes displayed excellent antioxidant activity and no cytotoxicity. The most potent antioxidant was found in the foliage, which also had a concentration-dependent detrimental effect on the viability of the three following cell lines: MCF-7, MDA-MB-231, and HepG-2, whereas roots and rhizomes had no impact [139]. In a recent study, the MTT assay measured the antiproliferative activity of *E. acoroides* metabolites, while the antioxidant activity was assessed by ABTS assay. This study showed that extracts can fight breast cancer by employing molecular docking to inhibit the HER2/EGFR/HIF-1*α* pathway. Additionally, the MTT assay showed that increasing the dosage of *E. acoroides* metabolites enhanced their morbidity in cancer cell lines. Furthermore, the investigation revealed that the secondary metabolites can operate as strong antioxidants, successfully obstructing an array of carcinogenic pathways [140]. Various tumor cell lines have shown anticancer activity in vitro when exposed to marine natural products. Furthermore, most reports on their mode of operation mediate through cancer cells’ lysis, necrosis, and apoptosis. Seagrass extracts demonstrate anticancer activity through their antiproliferative, antimetastatic, cytostatic, and cytotoxic, properties, as well as their capacity to induce apoptosis and antioxidative activity, induce cell-cycle arrest, restrict angiogenesis, and decrease cancer cell viability [141].

### 3.7. Anti-Inflammatory Potential

Inflammation is a complicated physiological reaction to various negative influences, defined by the recruitment and stimulation of immune cells (innate and adaptive immunity), which quickly regulate the stabilization and repair of injured body parts [142]. A recent study examined the anti-inflammatory potential of *Pasidonia oceanica* extract. The results showed that the high levels of COX-2 induced by LPS were reduced by the ethanolic extract of *P. oceanica*, suggesting an anti-inflammatory function linked to antioxidant effects. Furthermore, by modifying the intracellular cascades of ERK1/2 and Akt, *P. oceanica* can inhibit the NF-*κ*B signaling pathway, suggesting its anti-inflammatory potential [143]. A seagrass-derived fungus *Aspergillus insuetus* SYSU6925 produced one novel cyclohexenone derivative, two unexplained drimane sesquiterpenes, and seven additional known drimane sesquiterpenes. These metabolites were assessed for their anti-inflammatory activity, which revealed their anti-inflammatory solid properties by obstructing the creation of nitric oxide (NO) in cells under study [144].

### 3.8. Anti-Diabetic, Anti-Larval, Anti-Aging and Anti-Tumor Potential

Molecular visualizations using PyMOL and Discovery studio visualizer BIOVIA 2019 demonstrate that phytochemical substances found in seagrass *Enhalus acoroides* can block the action of glucosidase. This suggeststhat they could be employed as anti-diabetic therapeutics [145,146,147]. According to the research carried out by Ravikumar et al. [148], the phytochemical constituent in *Syringodium isoetifolium* extract is the least sensitive to fish pathogens. The methanolic extracts of seagrass *Thalassia hemprichii* leaves, rhizomes, and roots have larvicidal activity against *Aedes aegypti* 3rd instar larvae [91]. Cornara et al. [149] evaluated the anti-aging properties of seagrass *Posidonia oceanica*. As fibroblasts produce collagen, maintaining fibroblasts is necessary to slow down the aging process of the skin. Ethanolic extract *P. oceanica L. Delile* is a good candidate for the development of antiaging medications because it significantly increased the production of collagen in fibroblasts exposed to 5 and 10 µg/mL and increased lipolysis in the concentration limit of 10–200 µg/mL [149]. According to Abdelhameed et al. [71], *β*-sitosterol glucoside, 7*β*-hydroxy cholesterol, 7*β*-hydroxy sitosterol, stigmasterol glucoside, and TCC-1, a novel phytoceramide molecular species derived from the methylene dichloride–methanol fraction of *T. ciliatum*, exhibited cytotoxic effects with an IC_50_ value close to 20 μM against HepG2 and MCF7 cells. Another research work by Vani et al. [150] revealed that the crude extract of *Halophila beccarii* strongly suppresses α-amylase and α-glucosidase when considering the standard enzyme inhibitor acarbose. This study determined that the antidiabetic action was concentration-dependent, and the antihyperglycemic action was shown by inhibiting the carbohydrate digestive enzymes and glucose movement; consequently, the post-prandial hyperglycemia significantly dropped. *E. acoroides* develops anti-feedant, antibacterial, and antilarval secondary metabolites, which may serve as a chemical defense against predatory animals, competitors, and infectious agents [66].

According to the literature, the anti-inflammatory activity of methanol fraction from Seagrass *Zostera japonica* is reported [151]. According to a recent study, *Halophila stipulaceae* extract effectively treats diabetes-related oxidative stress and hyperlipidemia [152]. *Thalassia hemprichii* and *Cymodocea serrulata* and *C. serrulata* metabolites have strong antioxidant properties, while *H. uninervis* extract has a cytotoxic effect on human SKOV-3 ovarian and MCF-7 breast carcinoma cells [153]. The extract of seagrass, *H. ovalis*, collected from Pulicat Lake in Tamil Nadu, contains active principles such as kaempferol, p-hydroxy benzoic acid, t-cinnamic acid, syringic acid, catechin, and luteolin in significant amounts, which could be accountable for anticancer potential, providing an area for further experiments with isolated potential metabolites against breast cancer cells [154]. The effectiveness of a standardized polyphenol extract of seagrass *T. testudinum* (TTE) has been demonstrated in in vitro/in vivo using a syngeneic allograft murine colorectal cancer model and colon tumor cell lines (RKO, SW480, and CT26). Following the in vitro/in vivo findings, TTE treatment initiates autophagy stress pathways and ATF4-P53-NFκB specific gene expression. As a result, it encourages anti-tumor immunogenic cell death in vivo and suppresses colon cancer cell proliferation, motility, and angiogenesis pathways in vitro [155]. In a column chromatography study, the bioactive component of aqueous ethanol extract *Syringodium isoetifolium* was separated, and its anticancer properties were studied against hepatocellular carcinoma in vitro, in silico, and in vivo. The results show that the separated compound ‘phlorizin’ had good anticancer activity against hepatocellular carcinoma [156]. A recent investigation demonstrated that the bioderived TiO_2_ NPs could be an exciting candidate for medicinal purposes by highlighting the diverse attributes of *C. serrulata*-mediated TiO_2_ NPs, which included the antibacterial, antioxidant, and anti-biofilm inhibition effects that have minimal cell damage in nature [157]. Phytocompounds and the biomedical characteristics of seagrasses have been highlighted in the preceding sections. However, at least for many species, knowledge gaps regarding various aspects of seagrasses can be seen. Therefore, this review aims to identify the areas that require further investigation.

**Figure 11:**
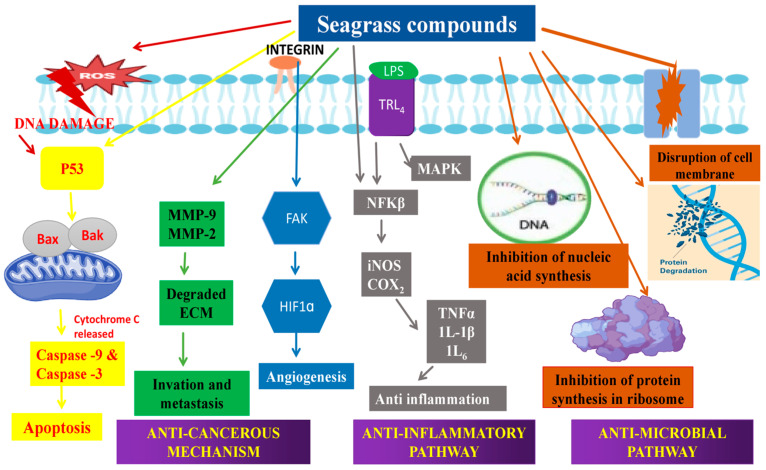
Mechanisms of bioactivities by seagrass compounds. Seagrass extracts and seagrass compounds show anticancer, anti-inflammatory, and antimicrobial activities.

Figure 11 shows the mechanisms of various bioactivities shown by seagrass extracts and their isolated compounds.

Figure 11 is a consolidation of the mechanism of anti-cancer activity [158,159], anti-inflammatory activity [160], and antimicrobial activity [161].

Other activities like antioxidant [162], and antidiabetic activities [163] were shown by these seagrass compounds which is well explained in the above sessions.

### 3.9. Bioprospecting Seagrass Microbiome

Numerous studies of endophytes of terrestrial plants have been conducted and provided a wide array of information regarding the diversity, distribution, and bioactive potential of endophytes. These endophytes were found to be a potential source of various unique bioactive molecules [164,165] and commercial enzymes [166], with characteristics that make them suitable for technological exploitation. On the other hand, comparatively little knowledge is available concerning endophytes of marine plants, such as seagrasses. According to some recent research, seagrasses are home to potential endophytes, which are beneficial for bioprospecting [167,168].

Compared to epiphytic microbes, endophytic microbes from seagrasses usually exhibited greater sensitivity against several human pathogenic microbes [169]. Research was carried out to evaluate the capability of the bacterial microbiome of seagrass *Enhalus* sp., from which 6 endophytes and 17 epiphytes were isolated; however, endophytes exhibited more significant biological activity than epiphytes against bacteria that form biofilms. Additionally, bacterial endophytes obstructed the growth of biofilm-forming bacteria than epiphytes [170].

Another study was executed to identify and determine the species of the endophytic bacteria that inhibit methicillin-resistant *Staphylococcus aureus* (MRSA) from the seagrasses of Rote Ndao, East Nusa Tenggara, Indonesia. The findings indicated that eight strains of endophytic bacteria isolated from different seagrasses *Enhalus acoroides*, *Cymodocea rotundata* and *Thalassia hemprichii*, in the Litianak and Oeseli Beaches, Rote, have antibacterial activity against MRSA [171]. Another study investigated the antibacterial activity of bacteria associated with seagrass from Indonesia’s North Java Sea against drug-resistant bacteria. This study aimed to examine the ability of bacteria associated with seagrasses—*E. acoroides*, *Syringodium* sp., *T. hemprichii*, and *Cymodocea* sp.—as sources of potential antibiotics. Two isolates showed antibacterial activity against bacteria resistant to multiple drugs [172]. In a recent investigation, the cultivable fungal group of the Baltic *Z. marina* was separated and recognized using a scientific method. Additionally, this study illustrates every fungal extract’s bioactivity and range of chemicals [173]. Another investigation was carried out to isolate and identify endophytic bacteria from seagrass, *T. hemprichii*, as producers of antibacterial agents. Some endophytic bacterial strains exhibited antibacterial properties against infectious bacteria like *S. aureus*, *Salmonella thyphi*, and *E. coli* [174]. A recent study found that endophytic fungi from *T. testudinum* exhibit potential bioactivity against *Labyrinthula* spp., a seagrass pathogenic organism, resulting in the death of plants [175].

The role of the epiphytic and endophytic microbes of the seagrass *Zostera marina* in pathogen elimination from the seagrass ecosystem was analyzed by Tasdemir et al. [176]. This study opens the door for more research to clarify the complex functions (as well as the mechanism) of the seagrass microbiome in preserving the well-being of coastal ecosystems and seagrass meadows.

### 3.10. Comprehensive Methodologies for Assessing Bioactivity in Seagrasses: Chemical, Biological, and Ecological Approaches

Various approaches are used to investigate the biochemical composition, biological activities, and ecological relationships of seagrasses through the study of bioactivity. Bioactive compounds, like antioxidants and antimicrobial agents, are identified and quantified using chemical analysis techniques like gas chromatography–mass spectrometry (GC-MS) and high-performance liquid chromatography (HPLC) [177,178]. The structural details of the compounds found in seagrass are further ascertained by nuclear magnetic resonance (NMR) spectroscopy [179]. The antioxidant capacity of seagrass extracts is evaluated by biological assays like DPPH and ABTS radical scavenging tests, while their efficacy against pathogens is assessed by antimicrobial assays [180]. The activity of seagrass extracts on cell viability is measured by cytotoxicity assays, such as MTT and LDH release tests [181]. By tracking growth patterns and environmental reactions, field research and remote sensing methods shed light on seagrass distribution, health, and ecological roles [182]. Molecular techniques that uncover genetic and metabolic pathways connected to bioactivity include metabolomics and DNA/RNA sequencing [183,184,185]. Finally, research on chemical ecology examines how chemical signaling functions in interactions between marine organisms and seagrasses [186,187,188,189,190]. When taken as a whole, these approaches provide a thorough knowledge of seagrass bioactivity and their ecological importance, which will, in turn, help the conservation strategies.

Comprehensive methods for determining the bioactivity of seagrasses use various analytical techniques, each with advantages and disadvantages. It usually involves a blend of various analytical methods [177,178]. The objectives of the study often influence the methodological choice. Gas chromatography–mass spectrometry (GC-MS) is a commonly used analytical tool for volatile and semi-volatile metabolites in metabolite profiling. It is less effective, nevertheless, with non-volatile or thermally unstable substances. It is not as effective in detecting polar metabolites, but it is especially helpful in profiling lipophilic compounds. Phenolic compounds and other secondary metabolites in seagrass extracts are separated and quantified using high-performance liquid chromatography (HPLC), frequently in conjunction with UV–Vis detection. It is beneficial when examining substances with unique UV absorbance characteristics. Integrated platforms that combine multiple analytical techniques—such as GC-MS, LC-MS, HPLC, and NMR—are frequently employed to accomplish thorough metabolite profiling. By enabling the simultaneous analysis of several metabolite classes, these platforms improve the scope and depth of profiling and enable the analysis of potential compounds found in the seagrass extracts.

## 4. Conclusions and Future Prospects

This article extensively reviews 183 research studies focused on the bioprospecting of seagrasses and their associated microbiomes. The reviewed literature encompasses a broad array of subjects, including the phytochemical and biochemical analysis of seagrasses, the assessment of seagrass bioactivities, and the exploration of the bioactive potential of seagrass-associated microbiomes. Using the available scientific literature, the authors of this article report biochemical and phytochemical constituents and pharmacological and other potent activities in various seagrasses. Since seagrasses are one of the most critical components of all marine habitats, studies focused on them have become increasingly important, as they are highly productive and serve as breeding grounds for several commercially important species. The biochemical contents of these plants can solve many human problems, such as several diseases, and have the potential for new inventions, such as natural antifoulants and UV sunscreens. This review touches on the critical points of the essential bioactive metabolites and seagrass’s antimicrobial and antioxidative properties in great depth. This review does not include an analysis of the literature on human clinical trials using bioactive molecules of seagrasses, as they form another vast area. However, several bioactive metabolites or compounds of seagrasses are still unknown, and researchers are working to discover all the essential compounds that aid human welfare. In the future, it will be essential to concentrate on isolating minor novel metabolites of potent therapeutic significance by utilizing innovative technologies to achieve good results.

The potential advantages of seagrass extracts in several disciplines, such as nutrition, environmental science, and skincare, have drawn attention. Seagrass extracts have several benefits over more conventional plant extracts, including the fact that they are a rich source of antioxidants, minerals (calcium, magnesium, and potassium), and vitamins (A, C, E, and K), a more environmentally responsible and sustainable choice than conventional plant extracts. It also possesses unique bioactive substances that are uncommon in terrestrial plants. Because of their adaptations, seagrasses can thrive in harsh marine environments with fluctuating salinities and reduced underwater lighting. This resilience may confer advantageous properties on seagrass extracts.

Despite the tremendous progress made in seagrass bioprospecting, some unanswered questions still need further investigation. First, more systematic research is required to understand the variety and functional roles of microbiomes associated with seagrasses, especially concerning their contributions to synthesizing bioactive compounds and ecosystem services. Furthermore, although a large number of research focuses on phytochemical and biochemical analyses, very little is known about how these discoveries relate to particular ecological roles or how environmental changes affect seagrass bioactivity. To better understand the possible uses of seagrasses, further research is needed to look into the metabolic and genetic pathways associated with the biosynthesis of bioactive compounds in seagrasses. Furthermore, the synergistic effects of combined seagrass species and their microbiomes on bioactivity have not received much attention. By encompassing these gaps, we may gain the best perspective possible on the potential applications of seagrasses and their microbiomes in environmental management and biotechnology.

## Figures and Tables

**Table 1:** Bioactive compounds in seagrasses (Astrix denotes the stereochemical configuration of the compound).

Compound Name	Seagrass	Bioactivity	References
Caffeic acid	*Posidonia oceanica*, *Zostera marina*, *Thalasia testudinum*, *Thalasia hemprichii*, *Thalassodendron ciliatum*	Antioxidant, Antiviral and Cytotoxic	[64,65]
Stigmasta-4,22-dien-6b-ol-3-one p-hydroxy-benzaldehyde Daucosterol Stigmast-22-en-3-one	*Enhalus acoroides*	Antifeedant	[66]
Stigmasta-4,22-dien-3,6-dione Hexacosyl alcohol Stigmasta-5,22-dien-3-O-b-dglucopyranoside	*Enhalus acoroides*	Antibacterial and antilarval activities	[66]
Deoxycymodienol Isocymodiene Nodosol Cymodienol (1S*,2S*,3S*,7R*,8S*,9R*,11R*,12S*,14R*)−7-bromo-tetradecahydro-12-hydroxy-1-isopropyl-8,12-dimethyl-4-methylene phenanthren-9,14-yl diacetate	*Cymodocea nodosa*	Antibacterial activity against methicillin-resistant (MRSA) strains	[67]
Coumaric acid	*Posidonia oceanica*, *Zostera marina*; *Thalassia testudinum*, *Halodule pinifolia*, *Thalassia hemprichii*	Antioxidant activity	[68]
Thalassiolins A (luteolin 7-O-ß-D-glucopyranosyl-2′-sulfate)	*Thalassia hemprichii* *Thalasia testudinum*	Anti-HIV activity and Antifouling activity	[56,69]
Thalassiolins B	*Thallasia testudinum*, *Thallasia hemprichii*	Anti-HIV activity, skin-regenerating activities and antioxidant	[56,69]
Thalassiolins C	*Thallasia testudinum*	Anti-HIV activity	[69]
Rutin Asebotin 3-hydroxyasebotin Quercetin-3-O-ß-d-xylopyranoside Catechin	*Thalassodendron ciliatum*	Antioxidant, Antiviral and Cytotoxicity against cancer cell lines	[70]
TCC-1 (10), 7β-hydroxy cholesterol 7β-hydroxysitosterol stigmasterol glucoside β-sitosterol glucoside	*Thalassodendron ciliatum*	Cytotoxic activity against HepG2 and MCF7 cells	[71]
Luteolin	*Halophila stipulacea*	Anticancerous activity	[72]
Quercetin	*Zostera marina* L., *Zostera noltii*	Antioxidant activity	[73]
Benzoic acid	*Thallasia testudinum* *Syringodium filiforme*	Antioxidant activity	[74]
Gallic acid	*Posidonia oceanica*	Radical scavenging activity	[75]
Chicoric acid	*Posidonia oceanica*	Antioxidant activity	[76]
Azelaic acid	*Syringodium filiforme* *Thalassia testudinum*	Antioxidant activity	[77]
Palmitoleic acid	*Syringodium filiforme*	Anti-inflammatory activity	[78]
Tricosane	*Syringodium filiforme*	Antibacterial	[79]
Oleic acid Linolenic acid Myristic acid Palmitic acid	*Halophila ovalis*	Antibacterial	[80]
Phytol	*Thalassia hemprichii* *Enhalus acoroides* *Cymodocea serrulata* *Cymodocea rotundata*	Antibacterial	[81]
Lyngbyabellin A	*Halophila stipulacea*	Anticancerous	[72]
Heptacosane	*Enhalus acoroides*	Antioxidant	[82]
Lauric acid	*Syringodium filiforme* *Thalassia testudinum*	Antibacterial	[83]
Octanoic acid	*Syringodium filiforme* *Thalassia testudinum*	Antibacterial	[84]

**Table 2:** Bioactivity of seagrass.

Solvent	Seagrass Species	Biological Activity	Reference
Chloroform, ethyl acetate, ethanol and hexane	*Halophila ovalis*, *Cymodocea serrulata*, *Halodule pinifolia*	Antimicrobial	[85]
Ethanol, methanol	*Halodule pinifolia*, *Cymodocea serrulata*	Antimicrobial	[86]
Methanol	*Halophila ovalis*	Antimicrobial	[58]
Aqueous ethanol	*Syringodium isoetifolium*, *Cymodocea serrulata*, *Halophila beccarii*	Larvicidal	[87]
Pectin	*Zostera marina*, *Phyllospadix iwatensis*	Cerium binding activity	[88]
Methanol	*Halodule pinifolia*, *Halophila ovalis*, *Syringodium isoetifolium*, *Thallasia hemprichii*, *Cymodocea serrulata*	Antioxidant	[89]
Ethanol	*Enhalus acoroides*, *Halophila ovalis*, *Halophila ovata*, *Halophila stipulacea*, *Thalassia hemprichii*, *Syringodium isoetifolium*, *Cymodocea serrulata*, *Halodule pinifolia*	Antibacterial	[49]
Methanol	*Syringodium isoetifolium*	Antioxidant	[43]
Sulfated polysaccharide	*Cymodocea nodosa*	Antioxidant, antimicrobial and cytotoxic properties	[90]
Methanol	*Thalassia hemprichii*	Larvicidal	[91]
n-hexane	*Thalassia hemprichii*, and *Enhalus acoroides*	Antifouling	[92]
Chloroform	*Posidonia oceanica*	Antimicrobial	[93]
Hydroalcoholic	*Syringodium isoetifolium*	Antibacterial, antifungal, antimicrobial, antifouling and anticancerous	[94]
Ethanol	*Cymodocea serrulata*, *Syringodium isoetifolium* and *Enhalus acoroides*	Nutritional supplements	[95]
70% Acetone	*Cymodocea rotundata* and *Cymodocea serrulata*	Antimicrobial, antifouling and antioxidant	[96]
Methanol	*Enhalus acoroides* and *Halophila ovalis*	Antifungal	[46]
MeOH/H_2_O(7:3)	*Posidonia oceanica*	Antiviral (against H5N1)	[97]
Methanol	*Thalassia hemprichii*	Antiviral	[98]
Aqueous EtOH	*Posidonia oceanica*	Antioxidant and antimicrobial	[99]
Ethyl acetate	*Posidonia oceanica*	Antimicrobial	[100]
Hexane and methanol	*Halophila stipulacea*	Cytotoxic activity, lipid-reducing activity, and antifouling activity.	[72]
85% Ethanol	*Thalassodendron ciliatum*	Antioxidant and hapatoprotective activity	[101]
EtOH/H_2_O (7:3)	*Posidonia oceanica*	Anticancerous	[102]
Chloroform	*Syringodium filiforme*	Anticancerous	[103]
Chloroform	*Thalassia testudinum*	Anticancerous	[104]
50% Ethanol	*Syringodium isoetifolium*	Anti-inflammatory	[105]
Ethanol	*Enhalus acoroides*, *Thalassia hemprichii*, and *Halophila ovalis.*	Antioxidant	[106]
Acetic acid	*Posidonia oceanica*	Antimicrobial	[107]
Water	*Posidonia oceanica*	Anticancerous	[108]
Acetic acid	*Posidonia oceanica*	Antimicrobial and anticancerous	[109]
96% Ethanol	*Cymodocea Rotundata*	Antibacterial	[110]
70% Ethanol	*Syringodium isoetifolium*	Anticancerous	[111]
Ethyl acetate	*Cymodocea serrulata*	Larvicidal	[112]
Water	*Posidonia oceanica*	Antifungal	[113]
Hydroalcoholic	*Enhalus acoroides*	Antioxidant	[82]
Ethanol	*Thalassia hemprichii* and *Halophila ovalis*	Antioxidant	[114]
Ethanol	*Enhalus acoroides*	Antioxidant	[115]
Water, alcohol, hydro alcohol, acetone and n-hexane.	*Syringodium isoetifolium*	Antioxidant	[116]
Ethanol	*Cymodocea serrulata*	Antidiabetic, anticancerous and antioxidant activity	[117]
Ethyl acetate	*Cymodocea serrulata*	Antibacterial and antioxidant	[118]
Ethanol	*Thalassia hemprichii* and *Zostera marina*	Antioxidant and antiobesity activity	[119]
Hexane, petroleum ether and acetone	*Ruppia cirrhosa*	Antioxidant and antimicrobial	[120]
70% ethanol	*Posidonia oceanica*	Antimicrobial activity	[121]
Ethyl acetate	*Halodule pinifolia*	Antioxidant activity	[122]
Methanol	*Posidonia oceanica*	Antioxidant activity and anticancerous activity	[123]
Ethyl acetate, hexane and methanol	*Halophila stipulacea*	Antioxidant, antimicrobial and analgesic activity	[124]

## Data Availability

No new data were created or analyzed in this study. Data sharing is not applicable to this article.

## Abbreviations

ODS column	Octadecyl-silica column
MIC	Minimum inhibitory concentration
DPPH	2,2-diphenyl-1-picryl-hydrazyl-hydrate
MECS	Methanolic extract of *Cymodocea serrulata*
AIDS	Acquired Immuno Deficiency Syndrome

## References

[1] Steele J.H., Beet A.R. (2003). Marine Protected Areas in “Nonlinear” Ecosystems. Proc. R. Soc. B Biol. Sci..

[2] The Oxygen Pumps in the Sea Seagrasses the Wealth of India Raw Materials Series (a Wealth of Information on Plants, Animals and Minerals of India). https://niscpr.res.in/includes/images/wealthofindia/WoI-Extension-Bulletin-Seagrasses-Jan13.pdf.

[3] Duffy J.E., Benedetti-Cecchi L., Trinanes J., Muller-Karger F.E., Ambo-Rappe R., Boström C., Buschmann A.H., Byrnes J., Coles R.G., Creed J. (2019). Toward a Coordinated Global Observing System for Seagrasses and Marine Macroalgae. Front. Mar. Sci..

[4] Ruiz-Frau A., Gelcich S., Hendriks I.E., Duarte C.M., Marbà N. (2017). Current State of Seagrass Ecosystem Services: Research and Policy Integration. Ocean Coast. Manag..

[5] Papenbrock J. (2012). Highlights in Seagrasses’ Phylogeny, Physiology, and Metabolism: What Makes Them Special?. ISRN Bot..

[6] James R.K., Keyzer L.M., van de Velde S.J., Herman P.M.J., van Katwijk M.M., Bouma T.J. (2023). Climate Change Mitigation by Coral Reefs and Seagrass Beds at Risk: How Global Change Compromises Coastal Ecosystem Services. Sci. Total Environ..

[7] Reynolds L.K., Waycott M., McGlathery K.J., Orth R.J. (2016). Ecosystem Services Returned through Seagrass Restoration. Restor. Ecol..

[8] Raja S., Subhashini P., Thangaradjou T. (2016). Differential Methods of Localisation of Fungal Endophytes in the Seagrasses. Mycology.

[9] Venkatachalam A., Thirunavukkarasu N., Suryanarayanan T.S. (2015). Distribution and Diversity of Endophytes in Seagrasses. Fungal Ecol..

[10] Supaphon P., Phongpaichit S., Rukachaisirikul V., Sakayaroj J. (2013). Antimicrobial Potential of Endophytic Fungi Derived from Three Seagrass Species: *Cymodocea serrulata*, *Halophila ovalis* and *Thalassia hemprichii*. PLoS ONE.

[11] Sakayaroj J., Preedanon S., Supaphon O., Jones E.B.G., Phongpaichit S. (2010). Phylogenetic Diversity of Endophyte Assemblages Associated with the Tropical Seagrass *Enhalus acoroides* in Thailand. Fungal Divers..

[12] Patil R., Mallya R. (2023). A Mini Review on Biological Activities of Genus *Thalassia*: A Marine Seagrass. J. Pharmacogn. Phytochem..

[13] Hu G.-P., Yuan J., Sun L., She Z.-G., Wu J.-H., Lan X.-J., Zhu X., Lin Y.-C., Chen S.-P. (2011). Statistical Research on Marine Natural Products Based on Data Obtained between 1985 and 2008. Mar. Drugs.

[14] Harvey A.L., Edrada-Ebel R., Quinn R.J. (2015). The Re-Emergence of Natural Products for Drug Discovery in the Genomics Era. Nat. Rev. Drug Discov..

[15] Blunt J.W., Copp B.R., Munro M.H.G., Northcote P.T., Prinsep M.R. (2010). Marine Natural Products. Nat. Prod. Rep..

[16] Capon R.J. (2001). Marine bioprospecting—Trawling for treasure and pleasure. Eur. J. Org. Chem..

[17] de la Torre-Castro M., Rönnbäck P. (2004). Links between humans and seagrasses—An example from tropical East Africa. Ocean. Coast. Manag..

[18] Haddaway N.R., Page M.J., Pritchard C.C., McGuinness L.A. (2022). PRISMA2020: An R package and Shiny app for producing PRISMA 2020-compliant flow diagrams, with interactivity for optimised digital transparency and Open Synthesis. Campbell Syst. Rev..

[19] Burt D.B. (2001). Evolutionary Stasis, Constraint and Other Terminology Describing Evolutionary Patterns. Biol. J. Linn. Soc..

[20] Orth R.J., Carruthers T.J., Dennison W.C., Duarte C.M., Fourqurean J.W., Heck K.L., Williams S.L. (2006). A global crisis for seagrass ecosystems. Bioscience.

[21] Nordlund L.M., Jackson E.L., Nakaoka M., Samper-Villarreal J., Beca-Carretero P., Creed J.C. (2018). Seagrass Ecosystem Services—What’s Next?. Mar. Pollut. Bull..

[22] Mtwana Nordlund L., Koch E.W., Barbier E.B., Creed J.C. (2016). Seagrass Ecosystem Services and Their Variability across Genera and Geographical Regions. PLoS ONE.

[23] Hemminga M.A., Duarte C.M. (2008). Seagrass Ecology.

[24] Fourqurean J.W., Duarte C.M., Kennedy H., Marbà N., Holmer M., Mateo M.A., Apostolaki E.T., Kendrick G.A., Krause-Jensen D., McGlathery K.J. (2012). Seagrass Ecosystems as a Globally Significant Carbon Stock. Nat. Geosci..

[25] Gillanders B.M. (2007). Seagrasses, Fish, and Fisheries. Seagrasses: Biology, Ecology and Conservation.

[26] Lilley R.J., Unsworth R.K.F. (2014). Atlantic Cod (*Gadus morhua*) Benefits from the Availability of Seagrass (*Zostera marina*) Nursery Habitat. Glob. Ecol. Conserv..

[27] Bujang J.S., Zakaria M.H., Arshad A. (2006). Distribution and Significance of Seagrass Ecosystems in Malaysia. Aquat. Ecosyst. Health Manag..

[28] Maxwell P.S., Eklöf J.S., van Katwijk M.M., O’Brien K.R., de la Torre-Castro M., Boström C., van der Heide T. (2017). The fundamental role of ecological feedback mechanisms for the adaptive management of seagrass ecosystems—A review. Biol. Rev..

[29] Cullen-Unsworth L., Unsworth R. (2013). Seagrass Meadows, Ecosystem Services, and Sustainability. Environ. Sci. Policy Sustain. Dev..

[30] Khalifa S.A.M., Elias N., Farag M.A., Chen L., Saeed A., Hegazy M.-E.F., Moustafa M.S., Abd El-Wahed A., Al-Mousawi S.M., Musharraf S.G. (2019). Marine Natural Products: A Source of Novel Anticancer Drugs. Mar. Drugs.

[31] Prasad G., Ramesh M.V., Ramesh T., Thomas G.M. (2023). Changing profile of natural organic matter in groundwater of a Ramsar site in Kerala implications for sustainability. Case Stud. Chem. Environ. Eng..

[32] Gayathri N., Prasad G., Prabhakaran V., Priya V. (2024). Understanding the impact of microplastic contamination on soil quality and eco-toxicological risks in horticulture: A comprehensive review. Case Stud. Chem. Environ. Eng..

[33] Widiastuti E.L., Rima K., Busman H. (2021). Anticancer Potency of Seagrass *(Enhalus acoroides)* Methanol Extract in the *HeLa* Cervical Cancer Cell Culture. Advances in Engineering Research/Advances in Engineering Research.

[34] Shailaja V., Christina V., Mohanapriya C., Sneha P., Sundaram R.L., Magesh R., Doss C.G.P., Gnanambal K.M.E. (2019). A Natural Anticancer Pigment, Pheophytin A, from a Seagrass Acts as a High Affinity Human Mitochondrial Translocator Protein (TSPO) Ligand, in Silico, to Reduce Mitochondrial Membrane Potential (∆ψ) in Adenocarcinomic A549 Cells. Phytomedicine.

[35] Imran M., Talpur F.N., Jan M.I., Khan A., Khan I. (2007). Analysis of nutritional components of some wild edible plants. J. -Chem. Soc. Pak..

[36] Kim D.H., Mahomoodally M.F., Sadeer N.B., Seok P.G., Zengin G., Palaniveloo K., Khalil A.A., Rauf A., Rengasamy K.R. (2021). Nutritional and Bioactive Potential of Seagrasses: A Review. S. Afr. J. Bot..

[37] Cui L., Jiang Z., Huang X., Liu S., Wu Y. (2023). Identification of Food Sources in Tropical Seagrass Bed Food Web Using Triple Stable Isotopes and Fatty Acid Signatures. Front. Mar. Sci..

[38] Clores M.A. (2023). Food-Web Dynamics in Three Seagrass Systems in Caramoan Peninsula, Philippines. Environ. Ecol. Res..

[39] Rondevaldova J., Quiao M.A., Drabek O., Dajcl J., Dela Pena-Galanida G.D., Leopardas V.E., Kokoska L. (2023). Mineral Composition of Seaweeds and Seagrasses of the Philippines. Phycologia.

[40] Pradheeba M., Dilipan E., Nobi E.P., Thangaradjou T., Sivakumar K. (2011). Evaluation of seagrasses for their nutritional value. Indian J. Geo-Mar. Sci..

[41] Immaculate J.K., Lilly T.T., Patterson J. (2018). Macro and micro nutrients of seagrass species from Gulf of Mannar, India. MOJ Food Process Technol..

[42] Rengasamy R.R.K., Radjassegarin A., Perumal A. (2013). Seagrasses as Potential Source of Medicinal Food Ingredients: Nutritional Analysis and Multivariate Approach. Biomed. Prev. Nutr..

[43] Bharatharathna P., Santhanam P. (2019). Analyses of phytochemical, biochemical, pigments and antioxidant activity of seagrass *Syringodium isoetifolium*. J. Adv. Sci. Res..

[44] Ragupathi Raja Kannan R., Arumugam R., Thangaradjou T., Anantharaman P. (2013). Phytochemical Constituents, Antioxidant Properties and P-Coumaric Acid Analysis in Some Seagrasses. Food Res. Int..

[45] Bharathi N.P., Jayalakshmi M., Amudha P., Vanitha V. (2019). Phytochemical screening and in vitro antioxidant activity of the seagrass *Cymodocea serrulata*. Indian J. Geo-Mar. Sci..

[46] De Vincenti L., Glasenapp Y., Cattò C., Villa F., Cappitelli F., Papenbrock J. (2018). Hindering the Formation and Promoting the Dispersion of Medical Biofilms: Non-Lethal Effects of Seagrass Extracts. BMC Complement. Altern. Med..

[47] Baby L., Sankar T.V., Chandramohanakumar N. (2017). Changes in Phenolic Compounds in Seagrasses against Changes in the Ecosystem. J. Pharmacogn. Phytochem..

[48] Smadi A., Ciavatta M., Bitam F., Carbone M., Villani G., Gavagnin M. (2017). Prenylated Flavonoids and Phenolic Compounds from the Rhizomes of Marine Phanerogam *Cymodocea nodosa*. Planta Medica.

[49] Kannan R.R.R., Arumugam R., Anantharaman P. (2012). Chemical Composition and Antibacterial Activity of Indian Seagrasses against Urinary Tract Pathogens. Food Chem..

[50] Setyoningrum D., Yamindago A., Hikmah Julinda Sari S., Maftuch M. (2020). Phytochemical Analysis and in vitro Antibacterial Activities of Seagrass *Enhalus acoroides* against *Staphylococcus aureus*. Res. J. Life Sci..

[51] Pharmawati M., Wrasiati L.P. (2020). Phytochemical screening and FTIR spectroscopy on crude extract from *Enhalus acoroides* leaves. Malays. J. Anal. Sci..

[52] Noor N.M., Febriani D., Ali M. (2022). Seagrass of *Enhalus acoroides* as a Traditional Body Scrubs in Preventing Malarial Bites by Pahawang Island Community in Indonesia. IOP Conf. Ser. Earth Environ. Sci..

[53] Kim H.S., Park N.H., Suk H.Y., You S.G., Woo J.H. (2022). Identification of Polyphenol Substances (MP-1) from Seagrass, *Phyllospadix japonica* Makino. Korean J. Environ. Agric..

[54] Das D., Arulkumar A., Paramasivam S., Lopez-Santamarina A., del Carmen Mondragon A., Miranda Lopez J.M. (2023). Phytochemical Constituents, Antimicrobial Properties and Bioactivity of Marine Red Seaweed (*Kappaphycus alvarezii*) and Seagrass (*Cymodocea serrulata*). Foods.

[55] Windyaswari A.S., Purba J.P., Nurrahmah S.S., Ayu I.P., Imran Z., Amin A.A., Kurniawan F., Pratiwi N.T.M., Iswantari A. (2019). Phytochemical Profile of Sea Grass Extract (*Enhalus acoroides*): A New Marine Source from Ekas Bay, East Lombok. IOP Conf. Ser. Earth Environ. Sci..

[56] Qi S.-H., Huang L.-S., He F., Zhang S., Dong J.-D. (2012). Phytochemical and Chemotaxonomic Investigation of Seagrass *Thalassia hemprichii (Ehrenb.) Aschers* (Hydrocharitaceae). Biochem. Syst. Ecol..

[57] Bitam F., Ciavatta M.L., Carbone M., Manzo E., Mollo E., Gavagnin M. (2010). Chemical Analysis of Flavonoid Constituents of the Seagrass *Halophila stipulacea*: First Finding of Malonylated Derivatives in Marine Phanerogams. Biochem. Syst. Ecol..

[58] Yuvaraj N., Kanmani P., Satishkumar R., Paari A., Pattukumar V., Arul V. (2012). Seagrass as a potential source of natural antioxidant and anti-inflammatory agents. Pharm. Biol..

[59] Tangon E., Elvinia R.A., Jocelyn A.P., Kingpu O.A. (2021). Phytochemical Screening and proximate composition of the seagrass *Halodule pinifolia* of the coastal waters of Carmen, Agusan Del Norte, Philippines. Int. J. Mod. Pharm. Res..

[60] Nazar S., Ravikumar S., Williams G.P., Ali M.S., Suganthi P. (2009). Screening of Indian coastal plant extracts for larvicidal activity of *Culex quinquefasciatus*. Indian J. Sci. Technol..

[61] Grignon-Dubois M., Rezzonico B. (2015). Phenolic Fingerprint of the Seagrass *Posidonia oceanica* from Four Locations in the Mediterranean Sea: First Evidence for the Large Predominance of Chicoric Acid. Bot. Mar..

[62] Blunt J.W., Carroll A.R., Copp B.R., Davis R.A., Keyzers R.A., Prinsep M.R. (2018). Marine Natural Products. Nat. Prod. Rep..

[63] Kavitha D., Padmini R., Dhanaraju M.D., Gopi C., Thiyagarajan D., Veeramaneni A. (2023). *Syringodium isoetifolium* Fosters an Antioxidant Defense System, Modulates Glycolytic Enzymes and Protects Membrane Integrity in DEN-induced Hepatocellular Carcinoma in Albino Wistar Rats. Ind. J. Pharm. Edu. Res..

[64] Haznedaroglu M.Z., Zeybek U. (2007). HPLC Determination of Chicoric Acid in Leaves of *Posidonia oceanica*. Pharm. Biol..

[65] Nuissier G., Rezzonico B., Grignon-Dubois M. (2010). Chicoric Acid from *Syringodium filiforme*. Food Chem..

[66] Qi S.-H., Zhang S., Qian P.-Y., Wang B.-G. (2008). Antifeedant, Antibacterial, and Antilarval Compounds from the South China Sea Seagrass *Enhalus acoroides*. Bot. Mar..

[67] Kontiza I., Stavri M., Zloh M., Vagias C., Gibbons S., Roussis V. (2008). New Metabolites with Antibacterial Activity from the Marine Angiosperm *Cymodocea nodosa*. Tetrahedron.

[68] Kannan R.R.R., Arumugam R., Anantharaman P. (2010). Antibacterial Potential of Three Seagrasses against Human Pathogens. Asian Pac. J. Trop. Med..

[69] Rowley D.C., Hansen M.S.T., Rhodes D., Sotriffer C.A., Ni H., McCammon J.A., Bushman F.D., Fenical W. (2002). Thalassiolins A–C: New Marine-Derived Inhibitors of HIV CDNA Integrase. Bioorganic Med. Chem..

[70] Hamdy A.-H.A., Mettwally W.S.A., El Fotouh M.A., Rodriguez B., El-Dewany A.I., El-Toumy S.A.A., Hussein A.A. (2012). Bioactive Phenolic Compounds from the Egyptian Red Sea Seagrass *Thalassodendron ciliatum*. Z. Für Naturforschung C.

[71] Abdelhameed R.F., Ibrahim A.K., Yamada K., Ahmed S.A. (2018). Cytotoxic and Anti-Inflammatory Compounds from Red Sea Grass *Thalassodendron ciliatum*. Med. Chem. Res..

[72] Bel Mabrouk S., Reis M., Sousa M.L., Ribeiro T., Almeida J.R., Pereira S., Antunes J., Rosa F., Vasconcelos V., Achour L. (2020). The Marine Seagrass *Halophila stipulacea* as a Source of Bioactive Metabolites against Obesity and Biofouling. Mar. Drugs.

[73] Kjersti Hasle E. (2018). Analysis of Polyphenolic Content in Marine and Aquatic Angiosperms from Norwegian Coastal Waters. Ph.D. Thesis.

[74] Velika B., Kron I. (2012). Antioxidant Properties of Benzoic Acid Derivatives against Superoxide Radical. Free Radic. Antioxid..

[75] Gao J., Hu J., Hu D., Yang X. (2019). A Role of Gallic Acid in Oxidative Damage Diseases: A Comprehensive Review. Nat. Prod. Commun..

[76] Zduńska K., Dana A., Kolodziejczak A., Rotsztejn H. (2018). Antioxidant Properties of Ferulic Acid and Its Possible Application. Ski. Pharmacol. Physiol..

[77] Jones D.A. (2009). Rosacea, reactive oxygen species, and azelaic acid. J. Clin. Aesthetic Dermatol..

[78] Weimann E., Silva M.B.B., Murata G.M., Bortolon J.R., Dermargos A., Curi R., Hatanaka E. (2018). Topical Anti-Inflammatory Activity of Palmitoleic Acid Improves Wound Healing. PLoS ONE.

[79] Tao C., Wu J., Liu Y., Liu M., Yang R., Lv Z. (2017). Antimicrobial Activities of Bamboo (*Phyllostachys heterocycla Cv. Pubescens*) Leaf Essential Oil and Its Major Components. Eur. Food Res. Technol..

[80] Yoon B., Jackman J., Valle-González E., Cho N.-J. (2018). Antibacterial Free Fatty Acids and Monoglycerides: Biological Activities, Experimental Testing, and Therapeutic Applications. Int. J. Mol. Sci..

[81] Islam M.T., Ali E.S., Uddin S.J., Shaw S., Islam M.A., Ahmed M.I., Chandra Shill M., Karmakar U.K., Yarla N.S., Khan I.N. (2018). Phytol: A Review of Biomedical Activities. Food Chem. Toxicol..

[82] Ahmed N. (2022). Phytochemical screening, antioxidant potential, isolation and characterization of bioactive compound from *Enhalus acoroides*. J. Pharm. Negat. Results.

[83] Anzaku A.A., Akyala J.I., Juliet A., Obianuju E.C. (2017). Antibacterial Activity of Lauric Acid on Some Selected Clinical Isolates. Ann. Clin. Lab. Res..

[84] Zhang H., Dolan H.L., Ding Q., Wang S., Tikekar R.V. (2019). Antimicrobial Action of Octanoic Acid against *Escherichia coli O157:H7* during Washing of Baby Spinach and Grape Tomatoes. Food Res. Int..

[85] Sangeetha J., Asokan S. (2015). Antibacterial activity of different sea grass Extracts against some human eye pathogens. World J. Pharm. Sci..

[86] Prabhakaran S., Rajaram R., Balasubramanian V., Mathivanan K. (2012). Antifouling potentials of extracts from seaweeds, seagrasses and mangroves against primary biofilm forming bacteria. Asian Pac. J. Trop. Biomed..

[87] Ali M.S., Ravikumar S., Beula J.M. (2012). Bioactivity of Seagrass against the Dengue Fever Mosquito *Aedes aegypti* Larvae. Asian Pac. J. Trop. Biomed..

[88] Kolenchenko E.A., Khotimchenko M.Y., Khozhaenko E.V., Khotimchenko Y.S. (2012). Strontium Sorption by Pectins Isolated from the Sea Grasses *Zostera marina* and *Phyllospadix iwatensis*. Russ. J. Mar. Biol..

[89] Athiperumalsami T., Kumar V., Jesudass L.L. (2008). Survey and Phytochemical Analysis of Seagrasses in the Gulf of Mannar, Southeast Coast of India. Bot. Mar..

[90] Kolsi R.B.A., Gargouri B., Sassi S., Frikha D., Lassoued S., Belghith K. (2017). In Vitro Biological Properties and Health Benefits of a Novel Sulfated Polysaccharide Isolated from *Cymodocea nodosa*. Lipids Health Dis..

[91] Purnomo H.K., Handayani W., Yasman Y. Bioprospecting of Potential Seagrass *Thalassia hemprichii (Ehrenb. Ex Solms) Asch.* (Hydrocharitaceae) Extract from Pramuka Island against *Aedes aegypti* L. Larvae. Proceedings of the 3rd International Symposium on Current Progress in Mathematics and Sciences 2017 (ISCPMS2017).

[92] Bengen D.G., Khoeri M.M., Marhaeni B., Radjasa O.K., Sabdono A., Sudoyo H. (2011). Antifouling Activity of Bacterial Symbionts of Seagrasses against Marine Biofilm-Forming Bacteria. J. Environ. Prot..

[93] Hammami S., Ben Salem A., Ashour M.L., Cheriaa J., Graziano G., Mighri Z. (2013). A Novel Methylated Sesquiterpene from Seagrass *Posidonia oceanica* (L.) Delile. Nat. Prod. Res..

[94] Kalaivani P., Amudha P. (2021). Identification of Bioactive Components in the Hydroalcoholic Extract of *Syringodium isoetifolium* and Assessment of Its Biological Activity by Gas Chromatography—Masspectrometry. Int. J. Res. Pharm. Sci..

[95] Vijayalingam T.A., Rajesh N.V. (2019). Seagrasses as Potential Source of Fodder for Livestock: Complete Proximate and Gas Chromatography-Mass Spectrometry (GCMS) Analysis. Ann. Phytomedicine Int. J..

[96] Wisespongpand P., Khantavong A., Phothong P., Wanghom W. (2022). Antimicrobial, Antioxidant, and Antifouling Activity from Extracts of Aboveground and Belowground Parts of Seagrasses *Cymodocea rotundata* and *Cymodocea serrulata*. J. Fish. Environ..

[97] Farid M.M., Marzouk M.M., Hussein S.R., Elkhateeb A., Abdel-Hameed E.S. (2018). Comparative study of *Posidonia oceanica* L.: LC/ESI/MS analysis, cytotoxic activity and chemosystematic significance. J. Mater. Environ. Sci..

[98] Hawas U.W., Abou El-Kassem L.T. (2017). Thalassiolin D: A New Flavone O-Glucoside Sulphate from the Seagrass *Thalass*. Hemprichii. Nat. Prod. Res..

[99] Abdelmohsen M., Hassanein H.D., Hassan R.A., Abreu A.C., Simões M., Nazif N.M., Abou-Setta L.M. (2016). Phytochemical analysis, in vitro evaluation of antioxidant and antimicrobial activities of phenolic extracts from *Posidonia oceanica* (L.) Delile leaves. J. Chem. Pharm. Res..

[100] Alfattani A., Blanchet E., Da Silva J.O., Leoni S., Allard P., Queiroz E., Roy M., Chave J., Lami R., Perron K. (2017). Bioactive Potential and Role of Secondary Metabolites within the Microorganism Community of the Sea Grass *Posidonia oceanica*. Planta Medica Int. Open.

[101] Mettwally W.S., Ragab T.I., Hamdy A.-H.A., Helmy W.A., Hassan S.A. (2021). Preliminary Study on the Possible Impact of *Thalassodendron ciliatum* (Forss.) Den Hartog Acidic Polysaccharide Fractions against TAA Induced Liver Failure. Biomed. Pharmacother..

[102] Piazzini V., Vasarri M., Degl’Innocenti D., Guastini A., Barletta E., Salvatici M.C., Bergonzi M.C. (2019). Comparison of Chitosan Nanoparticles and Soluplus Micelles to Optimize the Bioactivity of *Posidonia oceanica* Extract on Human Neuroblastoma Cell Migration. Pharmaceutics.

[103] Delange D.M., Garcia K.G., Rivera Y.H., Suárez Y.A., Cuesta R.G., Riera-Romo M., Echemendia O., Dutra L.M., Almeida J.R.G.D.S., Pérez-Martínez D. (2020). Chemical Composition and Biological Potential of a Chloroform Fraction from the Leaves of Marine Plant *Syringodium filiforme Kützing*. Pharmacogn. Mag..

[104] Riera-Romo M., Marrero-Delange D., Hernandez-Balmaseda I., González K., Pérez-Martínez D., Manso A., Labrada M., Rodeiro I. (2018). Chemical Characterization and Cytotoxic Potential of a Chloroform Fraction Obtained from Marine Plant *Thalassia testudinum*. J. Chromatogr. Sep. Tech..

[105] Susilo B., Oktavianty O., Rahayu F., Handayani M.L.W., Rohim A. (2023). Potential Transformation of Seagrass (*Syringodium isoetifolium*) into a Bioactive Food Ingredient Using Different Extraction Techniques. F1000Research.

[106] Thinh P.D., Hang C.T.T., Trung D.T., Nguyen T.-D. (2023). Pectin from Three Vietnamese Seagrasses: Isolation, Characterization and Antioxidant Activity. Processes.

[107] Punginelli D., Vazzana M., Mauro M., Catania V., Arizza V., Schillaci D. (2021). Antimicrobial activity from Polypeptide-rich extracts of the Seagrass *Posidonia oceanica*. J. Biol. Res..

[108] Abruscato G., Chiarelli R., Lazzara V., Punginelli D., Sugár S., Mauro M., Librizzi M., Di Stefano V., Arizza V., Vizzini A. (2023). In Vitro Cytotoxic Effect of Aqueous Extracts from Leaves and Rhizomes of the Seagrass *Posidonia oceanica (L.) Delile* on HepG2 Liver Cancer Cells: Focus on Autophagy and Apoptosis. Biology.

[109] Punginelli D., Catania V., Abruscato G., Luparello C., Vazzana M., Mauro M., Cunsolo V., Saletti R., Di Francesco A., Arizza V. (2023). New Bioactive Peptides from the Mediterranean Seagrass *Posidonia oceanica* (L.) Delile and Their Impact on Antimicrobial Activity and Apoptosis of Human Cancer Cells. Int. J. Mol. Sci..

[110] Amiin M.K., Lahay A.F. (2023). Anti-Bacterial Effectiveness of *Cymodocea rotundata* Extract and Assay for Primary Bioactive Composition. J. Aquatropica Asia.

[111] Kalaivani P., Amudha P., Chandramohan A., Vidya R., Prabhaharan M., Sasikumar P., Albukhaty S., Sulaiman G.M., Abomughaid M.M., Abu-Alghayth M.H. (2023). Evaluation of Cytotoxic Activity of *Syringodium isoetifolium* against Human Breast Cancer Cell Line—An in Silico and in Vitro Study. Arab. J. Chem..

[112] Amutha V., Aiswarya D., Deepak P., Selvaraj R., Tamilselvan C., Perumal P., Balasubramani G. (2023). Toxicity Potential Evaluation of Ethyl Acetate Extract of *Cymodocea serrulata* against the Mosquito Vectors Vis-a-Vis Zebrafish Embryos and *Artemia salina* Cysts. S. Afr. J. Bot..

[113] Berfad M.A., Fahej M.A.S., Kumar A., Edrah S. (2015). Preliminary phytochemical and antifungal studies of seagrass, *Posidonia oceanica* obtained from Mediterranean Sea of Libya. Int. J. Sci. Res..

[114] Phandee S., Buapet P. (2018). Photosynthetic and Antioxidant Responses of the Tropical Intertidal Seagrasses *Halophila ovalis* and *Thalassia hemprichii* to Moderate and High Irradiances. Bot. Mar..

[115] Sitania M., Kakisina P., Nindatu M., Moniharapon M., Kunda R.M. Alveolar Performance of Mice (*Mus musculus*) Exposure to Cigarette Smoke After-Treatment of Ethanol Extract of Seagrass (*Enhalus acoroides*). Proceedings of the 7th International Conference on Basic Sciences 2021 (ICBS 2021).

[116] Kavitha D., Padmini R., Chandravadivelu G., Magharla D.D. (2022). Phytoconstituents Screening and Antioxidant Activity of *Syringodium isoetifolium* Leaf Extracts. Indian J. Pharm. Sci..

[117] Dilipan E., Sivaperumal P., Kamala K., Ramachandran M., Vivekanandhan P. (2023). Green Synthesis of Silver Nanoparticles Using Seagrass *Cymodocea serrulata* (R.Br.) Asch. & Magnus, Characterization, and Evaluation of Anticancer, Antioxidant, and Antiglycemic Index. Biotechnol. Appl. Biochem..

[118] Narayanan M., Chanthini A., Devarajan N., Saravanan M., Sabour A., Alshiekheid M., Chi N.T.L., Brindhadevi K. (2023). Antibacterial and Antioxidant Efficacy of Ethyl Acetate Extract of *Cymodocea serrulata* and Assess the Major Bioactive Components in the Extract Using GC-MS Analysis. Process Biochem..

[119] Wagey B.T., Gunawan W.B., Lasabuda R., Mayulu N., Al Mahira M.F.N., Lailossa D.G., Nurkolis F. (2023). New insight on antioxidants and anti-obesity properties of two Indonesian seagrass *Thalassia hemprichii* and *Zostera marina*: An integrated molecular docking simulation with in vitro study. F1000Research.

[120] Chaabani E., Rebey I.B., Wannes W.A., Ksouri R., Shili A. (2023). Variability on the Phytochemical Composition, Antioxidant and Antimicrobial Activities of *Ruppia cirrhosa* Extract Using Two Different Methods of Extraction. Avicenna J. Clin. Microbiol. Infect..

[121] Abd-Elraoof W.A., Tayel A.A., El-Far S.W., Mohamed O., Diab A.M., Abonama O.M., Assas M.A., Abdella A. (2023). Characterization and Antimicrobial Activity of a Chitosan-Selenium Nanocomposite Biosynthesized Using *Posidonia oceanica*. RSC Adv..

[122] Santoso J., Purwaningsih S., Ramadhan W., Noveliyana Y. (2023). Bioactive Compounds, Phenol content and antioxidants activity of tropical seagrass *Halodule pinifolia*. Coast. Ocean. J..

[123] Kevrekidou A., Assimopoulou A.N., Trachana V., Stagos D., Malea P. (2024). Antioxidant Activity, Inhibition of Intestinal Cancer Cell Growth and Polyphenolic Compounds of the Seagrass *Posidonia oceanica*’s Extracts from Living Plants and Beach Casts. Mar. Drugs.

[124] Mabrouk S.B., Grami B., Ayache S.B., Kacem A. (2024). Antioxidant, antimicrobial and analgesic activities of the invasive seagrass *Halophila stipulacea* leaf and stem extracts. Authorea Preprints.

[125] Magdy M.D., Mohammed, Hamdy A.-H.A., El-Fiky N.M., Mettwally W.S.A., El-Beih A.A., Kobayashi N. (2014). Anti-Influenza a Virus Activity of a New Dihydrochalcone Diglycoside Isolated from the Egyptian Seagrass *Thalassodendron ciliatum* (Forsk.) Den Hartog. Nat. Prod. Res..

[126] Kumar C.S., Sarada D.V.L., Gideon T.P., Rengasamy R. (2008). Antibacterial Activity of Three South Indian Seagrasses, *Cymodocea serrulata*, *Halophila ovalis* and *Zostera capensis*. World J. Microbiol. Biotechnol..

[127] Hawas U.W. (2014). A New 8-Hydroxy Flavone O-Xyloside Sulfate and Antibacterial Activity from the Egyptian Seagrass *Thalassia hemprichii*. Chem. Nat. Compd..

[128] Berfad M.A., Alnour T.M. (2014). Phytochemical analysis and antibacterial activity of the 5 different extract from the seagrasses *Posidonia oceanica*. J. Med. Plants Stud..

[129] Emmanuel Joshua Jebasingh S., Lakshmikandan M., Sivaraman K., Uthiralingam M. (2015). Assessment of Antibacterial, Antifungal Property and Purification of Bioactive Compounds from Seagrass, *Thalassia hemprichii*. Proc. Natl. Acad. Sci. India Sect. B Biol. Sci..

[130] Mayavu P., Sugesh S., Ravindran V.J. (2009). Antibacterial activity of seagrass species against biofilm forming bacteria. Res. J. Microbiol..

[131] Kumar S.R., Ramanathan G., Subhakaran M., Inbaneson S.J. (2009). Antimicrobial Compounds from Marine Halophytes for Silkworm Disease Treatment. Int. J. Med. Med. Sci..

[132] Krylova N.V., Leonova G.N., Maystrovskaya O.S., Popov A.M., Artyukov A.A. (2018). Mechanisms of Antiviral Activity of the Polyphenol Complex from Seagrass of the *Zosteraceae* Family against Tick-Borne Encephalitis Virus. Bull. Exp. Biol. Med..

[133] Santoso J., Anwariyah S., Rumiantin R.O., Putri A.P., Ukhty N., Yoshie-Stark Y. (2012). Phenol content, antioxidant activity and fibers profile of four tropical seagrasses from Indonesia. J. Coast. Dev..

[134] Jeyapragash D., Subhashini P., Raja S., Abirami K., Thangaradjou T. (2016). Evaluation of In-Vitro Antioxidant Activity of Seagrasses: Signals for Potential Alternate Source. Free Radic. Antioxid..

[135] Baehaki A., Widiastuti I., Nurul Jannah H. (2017). Antioxidant Activity of Extracts of *Halodule pinifolia* Seagrass from Solvents with Different Polarities. Orient. J. Chem..

[136] Girija K., Parthiban C., Hemalatha A., Saranya C., Anantharaman P. (2013). Evaluation of antioxidant activities and preliminary phytochemical analysis of seagrasses *Halodule pinifolia*, *Halophila ovalis* and *Syringodium isoetifolium*. J. Phytochem..

[137] Ramalingam N., Ramakrishnan K., Krishnan C., Sankar S. (2013). Phytochemical Analysis, HPTLC Finger Printing, in Vitro Antioxidant and Cytotoxic Activity of *Cymodocea serrulata*. Pharmacogn. J..

[138] Messina C.M., Arena R., Manuguerra S., Pericot Y., Curcuraci E., Kerninon F., Renda G., Hellio C., Santulli A. (2021). Antioxidant Bioactivity of Extracts from Beach Cast Leaves of *Posidonia oceanica* (L.) Delile. Mar. Drugs.

[139] El Shaffai A., Mettwally W.S.A., Mohamed S.I.A. (2023). A Comparative Study of the Bioavailability of Red Sea Seagrass, *Enhalus acoroides* (L.f.) Royle (Leaves, Roots, and Rhizomes) as Anticancer and Antioxidant with Preliminary Phytochemical Characterization Using HPLC, FT-IR, and UPLC-ESI-TOF-MS Spectroscopic Analysis. Beni-Suef Univ. J. Basic Appl. Sci..

[140] Prajoko Y.W., Qhabibi F.R., Gerardo T.S., Kizzandy K., Tanjaya K., Willyanto S.E., Permatasari H.K., Surya R., Mayulu N., Taslim N.A. (2024). Revealing Novel Source of Breast Cancer Inhibitors from Seagrass *Enhalus acoroides*: In Silico and in Vitro Studies. Molecules.

[141] Su S., Cheng X., Wink M. (2015). Cytotoxicity of Arctigenin and Matairesinol against the T-Cell Lymphoma Cell Line CCRF-CEM. J. Pharm. Pharmacol..

[142] Chen L., Deng H., Cui H., Fang J., Zuo Z., Deng J., Li Y., Wang X., Zhao L. (2018). Inflammatory Responses and Inflammation-Associated Diseases in Organs. Oncotarget.

[143] Vasarri M., Leri M., Barletta E., Ramazzotti M., Marzocchini R., Degl’Innocenti D. (2020). Anti-Inflammatory Properties of the Marine Plant *Posidonia oceanica* (L.) Delile. J. Ethnopharmacol..

[144] Hu Z., Chen J., Liu Q., Wu Q., Chen S., Wang J., Li J., Liu L., Gao Z. (2023). Cyclohexenone Derivative and Drimane Sesquiterpenes from the Seagrass-Derived Fungus *Aspergillus insuetus*. Chem. Biodivers..

[145] Al-Ansari M.M., Nora Dahmash Al-Dahmash G.K. (2023). Jhanani. Anti-Candida, Antioxidant and Antidiabetic Potential of Ethyl Acetate Extract Fraction-7a from *Cymodocea serrulata* and Its Bioactive Compound Characterization through FTIR and NMR. Environ. Res..

[146] Fatmawati Y., Sandrina S., Aina R.N., Narulita E. (2022). Molecular Docking Analysis of Seagrass (*Enhalus acoroides*) Phytochemical Compounds as an Antidiabetic. J. Biol. Res. Boll. Della Soc. Ital. Biol. Sper..

[147] Baehaki A., Herpandi H.H., Lestari S., Hendri M., Ariska F. (2020). Antidiabetic Activity with N-Hexane, Ethyl-Acetate and Ethanol Extract of *Halodule uninervis* Seagrass. Pharmacogn. J..

[148] Ravikumar S., Vinoth R., Selvan G.P. (2011). Bioactive Potential of a Seagrass *Syringodium isoetifolium* against Bacterial Fish Pathogens. J. Pharm. Res..

[149] Cornara L., Pastorino G., Borghesi B., Salis A., Clericuzio M., Marchetti C., Damonte G., Burlando B. (2018). *Posidonia oceanica* (L.) *Delile* Ethanolic Extract Modulates Cell Activities with Skin Health Applications. Mar. Drugs.

[150] Vani M., Vasavi T., Uma M.D.P. (2018). Evaluation of in Vitro Antidiabetic activity of Methanolic extract of Seagrass *Halophila beccarii*. Asian J. Pharm. Clin. Res..

[151] Hua K.-F., Hsu H.-Y., Su Y.-C., Lin I.-F., Yang S.-S., Chen Y.-M., Chao L.K. (2005). Study on the Antiinflammatory Activity of Methanol Extract from Seagrass *Zostera japonica*. J. Agric. Food Chem..

[152] Hegazi N.M., Saad H., Marzouk M.M., Abdel M.F., El H., Zayed A., Ulber R., Ezzat S.M. (2021). Molecular Networking Leveraging the Secondary Metabolomes Space of *Halophila stipulaceae (Forsk.) Aschers.* And *Thalassia hemprichii (Ehrenb. Ex Solms) Asch*. In Tandem with Their Chemosystematics and Antidiabetic Potentials. Mar. Drugs.

[153] Ghandourah M., Hawas U.W., Abou El-Kassem L.T., Shaher F.M. (2020). Fatty Acids and Other Chemical Compositions of Some Seagrasses Collected from the Saudi Red Sea with Potential of Antioxidant and Anticancer Agents. Thalass. Int. J. Mar. Sci..

[154] Sharma H., Stephen N.M., Gopal S.S., Udayawara Rudresh D., Kavalappa Y.P., Haranahalli Shivarudrappa A., Gavirangappa H., Ponesakki G. (2020). Phenolic Extract of Seagrass, *Halophila ovalis* Activates Intrinsic Pathway of Apoptosis in Human Breast Cancer (MCF-7) Cells. Nutr. Cancer.

[155] Hernández-Balmaseda I., Guerra I.R., Declerck K., Isidrón J.A.H., Pérez-Novo C., Van Camp G., De Wever O., González K., Labrada M., Carr A. (2021). Marine Seagrass Extract of *Thalassia testudinum* Suppresses Colorectal Tumor Growth, Motility and Angiogenesis by Autophagic Stress and Immunogenic Cell Death Pathways. Mar. Drugs.

[156] PPerumal P., Arthanari U., Sanniyasi E. (2023). Phlorizin Isolated from Seagrass *Syringodium isoetifolium* Inhibits Diethylnitrosamine and Carbon Tetrachloride-Induced Hepatocellular Carcinoma in BALB/c Mice. S. Afr. J. Bot..

[157] Narayanan M., Srinivasan S., Gnanasekaran C., Ramachandran G., Chelliah C.K., Rajivgandhi G., Maruthupandy M., Quero F., Li W.-J., Hayder G. (2024). Synthesis and Characterization of Marine Seagrass (*Cymodocea serrulata*) Mediated Titanium Dioxide Nanoparticles for Antibacterial, Antibiofilm and Antioxidant Properties. Microb. Pathog..

[158] Hemmati S., Noveiry B.B., Keshavarz-Fathi M. (2021). Cancer and Allergy; Molecular Association and Integrated Therapies. Immunol. Genet. J..

[159] Bushehri R.H., Navabi P., Saeedifar A.M., Keshavarzian N., Rouzbahani N.H., Mosayebi G., Ghazavi A., Ghorban K., Ganji A. (2023). Integration of phytotherapy and chemotherapy: Recent advances in anticancer molecular pathways. Iran. J. Basic Med. Sci..

[160] Mohammadi A., Rahbardar M.G., Hosseinzadeh H. (2023). Antidotal and protective effects of mangosteen (*Garcinia mangostana*) against natural and chemical toxicities: A review. Iran. J. Basic Med. Sci..

[161] Bertrand B., Morales-Martínez A., Hernández-Adame P.L., Muñoz-Garay C. (2023). Multirresistencia antibióticos y alternativas para resolver esta crisis. Rev. Digit. Univ..

[162] Varshney M., Kumar B., Rana V.S., Sethiya N.K. (2023). An overview on therapeutic and medicinal potential of poly-hydroxy flavone viz. Heptamethoxyflavone, Kaempferitrin, Vitexin and Amentoflavone for management of Alzheimer’s and Parkinson’s diseases: A critical analysis on mechanistic insight. Crit. Rev. Food Sci. Nutr..

[163] Javadi B., Sobhani Z. (2024). Role of apigenin in targeting metabolic syndrome: A systematic review. Iran. J. Basic Med. Sci..

[164] Suryanarayanan T.S., Thirunavukkarasu N., Govindarajulu M.B., Sasse F., Jansen R., Murali T.S. (2009). Fungal Endophytes and Bioprospecting. Fungal Biol. Rev..

[165] Nair A.M., Yesodharan G., Arun K., Prasad G. (2023). Unveiling the factors influencing groundwater resources in a coastal environment-a review. Agron. Res..

[166] Suryanarayanan T.S., Thirunavukkarasu N., Govindarajulu M.B., Gopalan V. (2012). Fungal Endophytes: An Untapped Source of Biocatalysts. Fungal Divers..

[167] Supaphon P., Phongpaichit S., Sakayaroj J., Rukachaisirikul V., Kobmoo N., Spatafora J.W. (2017). Phylogenetic Community Structure of Fungal Endophytes in Seagrass Species. Bot. Mar..

[168] Prasad G., Mamane H., Ramesh M.V. (2022). Geogenic and anthropogenic contamination of groundwater in a fragile eco-friendly region of southern Kerala, India. Agron. Res..

[169] Ravikumar S., Thajuddin N., Suganthi P., Jacob Inbaneson S., Vinodkumar T. (2010). Bioactive potential of seagrass bacteria against human bacterial pathogens. J. Environ. Biol..

[170] Marhaeni B., Radjasa O.K., Bengen D.G., Kaswadji R.F. (2024). Screening of Bacterial Symbionts of Seagrass *Enhalus* Sp. against biofilm-forming bacteria. J. Coast. Dev..

[171] Fitri D.S., Pangastuti A., Susilowati A., Sutarno S. (2017). Endophytic Bacteria Producing Antibacterial against Methicillinresistant *Staphylococcus aureus* (MRSA) in Seagrass from Rote Ndao, East Nusa Tenggara, Indonesia. Biodiversitas J. Biol. Divers..

[172] Cristianawati O., Sibero M.T., Ayuningrum D., Nuryadi H., Syafitri E., Riniarsih I.O., Radjasa O.K. (2019). Screening of antibacterial activity of seagrass-associated bacteria from the North Java Sea, Indonesia against multidrug-resistant bacteria. AACL Bioflux.

[173] Petersen L.-E., Marner M., Labes A., Tasdemir D. (2019). Rapid Metabolome and Bioactivity Profiling of Fungi Associated with the Leaf and Rhizosphere of the Baltic Seagrass *Zostera marina*. Mar. Drugs.

[174] Ginting E.L., Maarisit I., Kemer K., Siby M.S., Tilaar S.O., Moko E.M., Tumbol R.A. (2023). Isolation and Identification of Endophytic Bacteria from Seagrass *Thalassia hemprichii* as Antibacterial Producer. Appl. Biochem. Microbiol..

[175] Ugarelli K., Jagels A., Choi C.J., Loesgen S., Stingl U. (2024). Fungal Endophytes from *Thalassia testudinum* Show Bioactivity against the Seagrass Pathogen, *Labyrinthula* spp.. Front. Mar. Sci..

[176] Tasdemir D., Scarpato S., Utermann-Thüsing C., Jensen T., Blümel M., Wenzel-Storjohann A., Welsch C., Echelmeyer V.A. (2023). Epiphytic and Endophytic Microbiome of the Seagrass *Zostera marina*: Do They Contribute to Pathogen Reduction in Seawater?. Sci. Total. Environ..

[177] Goda M., Eltamany E.E., Habib E.S., Hassanean H., Ahmed S.A.E., Abdelhameed R.F., Ibrahim A.K. (2020). B:Gas Chromatography-Mass Spectrometry Analysis of Marine Seagrass *Thalassodendron ciliatum* Collected from Red Sea. Rec. Pharm. Biomed. Sci./Rec. Pharm. Biomed. Sci..

[178] Menaa F., Wijesinghe U., Thiripuranathar G., Althobaiti N.A., Albalawi A.E., Khan B.A., Menaa B. (2021). Marine Algae-Derived Bioactive Compounds: A New Wave of Nanodrugs?. Mar. Drugs.

[179] Grauso L., Li Y., Scarpato S., Shulha O., Rárová L., Strnad M., Teta R., Mangoni A., Zidorn C. (2019). Structure and Conformation of Zosteraphenols, Tetracyclic Diarylheptanoids from the Seagrass *Zostera marina*: An NMR and DFT Study. Org. Lett..

[180] Sarvesh N., Afeeza K., Suresh V., Dilipan E. (2024). Development of the Antioxidant Property of Seagrass Extract-Based Hydrogel for Dental Application. Cureus.

[181] Grauso L., Li Y., Scarpato S., Cacciola N.A., De Cicco P., Zidorn C., Mangoni A. (2022). A Cytotoxic Heterodimeric Cyclic Diarylheptanoid with a Rearranged Benzene Ring from the Seagrass *Zostera marina*. J. Nat. Prod..

[182] Veettil B.K., Ward R.D., Lima M.D.A.C., Stankovic M., Hoai P.N., Quang N.X. (2020). Opportunities for Seagrass Research Derived from Remote Sensing: A Review of Current Methods. Ecol. Indic..

[183] Davey P.A., Pernice M., Sablok G., Larkum A., Lee H.T., Golicz A., Edwards D., Dolferus R., Ralph P. (2016). The Emergence of Molecular Profiling and Omics Techniques in Seagrass Biology; Furthering Our Understanding of Seagrasses. Funct. Integr. Genom..

[184] Kumar M., Kuzhiumparambil U., Pernice M., Jiang Z., Ralph P.J. (2016). Metabolomics: An Emerging Frontier of Systems Biology in Marine Macrophytes. Algal Res..

[185] Griffiths L.L., Melvin S.D., Connolly R.M., Pearson R.M., Brown C.J. (2020). Metabolomic Indicators for Low-Light Stress in Seagrass. Ecol. Indic..

[186] Arnold T.M., Targett N.M. (2002). Marine tannins: The importance of a mechanistic framework for predicting ecological roles. J. Chem. Ecol..

[187] Paul V.J., Arthur K.E., Ritson-Williams R., Ross C., Sharp K. (2007). Chemical Defenses: From Compounds to Communities. Biol. Bull..

[188] Sieg R.D., Kubanek J. (2013). Chemical Ecology of Marine Angiosperms: Opportunities at the Interface of Marine and Terrestrial Systems. J. Chem. Ecol..

[189] Zupo V., Maibam C., Buia M.C., Gambi M.C., Patti F.P., Scipione M.B., Lorenti M., Fink P. (2015). Chemoreception of the Seagrass *Posidonia oceanica* by Benthic Invertebrates Is Altered by Seawater Acidification. J. Chem. Ecol..

[190] Mutalipassi M., Fink P., Maibam C., Porzio L., Buia M.C., Gambi M.C., Patti F.P., Scipione M.B., Lorenti M., Zupo V. (2020). Ocean Acidification Alters the Responses of Invertebrates to Wound-Activated Infochemicals Produced by Epiphytes of the Seagrass *Posidonia oceanica*. J. Exp. Mar. Biol. Ecol..

